# Iron stimulates greenhouse gas production in Southeast Asian peatlands

**DOI:** 10.1093/ismeco/ycag139

**Published:** 2026-06-05

**Authors:** Andrew Elohim Laloo, Abhishek Gupta, Valeria Verrone, Rama Kant Dubey, Pierre Taillardat, Rajpirathap Nagasaijanani, Muhammad Iman Faisal Harahap, Agnieszka M Banas, Krzysztof Banas, Raktim Bhattacharya, Sophie Lok Sze Min, Yi Zi Koh, Fairuz Razali, Nikken Irfa Nastiti, Eka Sri Kandi Putri, Sofyan Kurnianto, Chandra Shekhar Deshmukh, Romy Chakraborty, Sanjay Swarup

**Affiliations:** National University of Singapore Environmental Research Institute, National University of Singapore, Singapore 117411, Singapore; Singapore Centre for Environmental Life Sciences Engineering,, Singapore 637551, Singapore; National University of Singapore Environmental Research Institute, National University of Singapore, Singapore 117411, Singapore; Singapore Centre for Environmental Life Sciences Engineering,, Singapore 637551, Singapore; National University of Singapore Environmental Research Institute, National University of Singapore, Singapore 117411, Singapore; National University of Singapore Environmental Research Institute, National University of Singapore, Singapore 117411, Singapore; Department of Biological Sciences, National University of Singapore, Singapore 117558, Singapore; National University of Singapore Environmental Research Institute, National University of Singapore, Singapore 117411, Singapore; Asian School of the Environment, Nanyang Technological University, Singapore 639798, Singapore; Singapore Centre for Environmental Life Sciences Engineering,, Singapore 637551, Singapore; Asia Pacific Resources International Ltd. Pelalawan Regency, Riau 28300, Indonesia; Singapore Synchrotron Light Source, National University of Singapore, 5 Research Link, Singapore 117603, Singapore; Singapore Synchrotron Light Source, National University of Singapore, 5 Research Link, Singapore 117603, Singapore; National University of Singapore Environmental Research Institute, National University of Singapore, Singapore 117411, Singapore; National University of Singapore Environmental Research Institute, National University of Singapore, Singapore 117411, Singapore; Singapore Centre for Environmental Life Sciences Engineering,, Singapore 637551, Singapore; National University of Singapore Environmental Research Institute, National University of Singapore, Singapore 117411, Singapore; Department of Silviculture, IPB University, Bogor, West Java 16680, Indonesia; Department of Silviculture, IPB University, Bogor, West Java 16680, Indonesia; Asia Pacific Resources International Ltd. Pelalawan Regency, Riau 28300, Indonesia; Asia Pacific Resources International Ltd. Pelalawan Regency, Riau 28300, Indonesia; Department of Ecology, Earth and Environmental Sciences Area, Lawrence Berkeley National Laboratory, Berkeley, CA 94720, United States; National University of Singapore Environmental Research Institute, National University of Singapore, Singapore 117411, Singapore; Singapore Centre for Environmental Life Sciences Engineering,, Singapore 637551, Singapore; Department of Biological Sciences, National University of Singapore, Singapore 117558, Singapore

**Keywords:** greenhouse gas, peat microbiome, iron reduction, methanogenesis, fermentation, syntrophy

## Abstract

Iron (Fe) acts as a terminal electron acceptor for microbes under oxygen-limiting conditions, leading to organic matter decomposition and emission of carbon greenhouse gases (C-GHG) in peatlands. The underlying molecular mechanism governing this phenomenon is not fully understood. Here, we investigate the role of Fe(III) in C-GHG production using a multiomics approach through a lab-based microcosm experiment on tropical peat collected from three different depths of an *Acacia* plantation site. Fe(III) amendment leads to severalfold higher production of CO_2_ and CH_4_ than unamended setups. Moreover, the gas profile correlates with depletion of carbohydrate compounds in the upper and middle depths, indicating microbial preference for readily fermentable substrates, aligning with increased CO_2_ production as well as the enrichment of fermentative microbes and genes associated with complex organic carbon degradation. Our study highlights the underestimated role of Fe(III) in carbon transformation and its potential to exacerbate climate change impacts on tropical peatlands.

## Introduction

Global peatlands cover over 500 million hectares and are estimated to store ~604 ± 130 gigatonnes of carbon (GtC) [1, 2]. Southeast Asian peatlands cover 25 million hectares and are estimated to store 36–108 GtC [3, 4]. Despite being one of the largest terrestrial carbon sinks [5–7], peatlands are under threat due to increasing land demands driven by population growth and economic development [8–10]. Moreover, shifts in hydrology due to land-use change and drainage reverse the role of peatlands from a carbon sink to a source of carbon greenhouse gases (C-GHG) [11, 12].

Peatlands have accumulated carbon over a millennia timescale due to the slow decomposition rates of deposited plant materials under waterlogged conditions [13]. Peat is composed of a heterogeneous complex of organic molecules, from highly recalcitrant to easily metabolized compounds [14, 15]. Nearly 70% of organic matter (OM) degradation in peatlands is primarily through heterotrophic respiration and to a lower extent, photochemical degradation of dissolved OM [16, 17]. Under anoxic conditions, microbes decompose larger molecules to organic polymers, oligomers, and monomers, which are then fermented to yield a narrow range of fermentation products such as alcohols and acetate. These compounds ultimately get oxidized to CO_2_ by microbes with the help of different terminal electron acceptors (TEAs) [18, 19]. Recent studies showed that amendment of TEAs such as NO_3_^−^ and SO_4_^2−^ in peat systems enhanced CO_2_ production [20–22]. However, in peat lacking inorganic TEAs, OM itself can serve as a TEA under anoxic conditions, leading to the formation of CO_2_ and CH_4_ [19, 23]. Additionally, in anaerobic conditions methanogenic archaea convert CO_2_ and acetate/hydrogen to CH_4_ through acetoclastic and hydrogenotrophic methanogenesis [19, 24]. This generated CH_4_ is oxidized to CO_2_ by methanotrophs coupled to TEAs [25, 26]. Acetoclastic methanogenesis yields isotopically heavier δ^13^C-CH_4_ (−65% to −50%) while hydrogenotrophic methanogenesis produces lighter δ^13^C-CH_4_ (−110% to −60%) [27, 28]. Hence, it can provide additional insights into the metabolic pathways, microbial activity, and environmental conditions in peatlands. A study by Moore and Dalva (1997) showed that in temperate peatlands, despite low availability of TEAs, CO_2_ production was severalfold higher than CH_4_ indicating that stoichiometry of methanogenesis seems to be different from the normal 1:1 ratio of CO_2_ to CH_4_ production [29]. This suggests that alternative terminal electron accepting pathways are operating in peatlands [23, 30] supporting higher CO_2_ production. Recently, Duchesneau *et al*., suggested that TEAs bound to OM are released under microbial degradation processes [23]. Studies on TEAs in tropical peatlands are limited, and their roles have never been studied in relation to microbially mediated C-GHG production. Thus, investigating the role of TEAs in tropical peatlands is central to the fundamental understanding of C-GHG production and carbon source-sink relationships [31].

The biogeochemical cycling of carbon has been well documented to be connected to the redox cycling of iron [32]. Several exploratory studies show that iron-cycling microbes are widespread in peatlands suggesting they play a significant role in influencing carbon cycling and C-GHG emissions [22, 33–35]. Romanowicz *et al*. [36] showed genomic evidence in thawing permafrost that microbial carbon degradation was dominated by iron redox [37]. Additionally, in tropical peatlands iron has been shown to be directly correlated to photo-mineralization of dissolved organic carbon in canals, leading to increased CO_2_ production [38]. Iron (Fe) is a redox-active element and is known to play a dual role in OM stabilization and degradation in peat and other natural environments by acting as a TEA [39, 40]. Fe in peatlands originates from atmospheric, geological, hydrological, and biological processes [41, 42]. The majority of Fe is bound to OM as a “rusty sink” which upon release is one of the most preferred TEAs by microbes under anoxic/suboxic conditions, thereby stimulating OM degradation [40]. Arising from the dual role of Fe in OM stabilization and degradation, several studies have investigated the outcome of such degradation on C-GHG production. For instance, (semi)conductive Fe minerals influenced CH_4_ production in sediment, soil, and anaerobic digesters by acting as an electron transfer conduit between Fe reducers and methanogens [43–46]. Fe reducers could access protected carbon in peat systems, thereby making carbon available for substrate utilization [47]. In subarctic permafrost and temperate peatlands, reactive Fe promotes the production of CO_2_ and CH_4_ potentially releasing protected carbon [22, 47, 48]. As tropical peatlands have different peat chemistry and TEA availability, the mechanisms underlying the release of CO_2_ and CH_4_ could be different [49]. Although organic-rich peat contains low total Fe [50, 51], disturbed or agriculturally converted peatlands exhibit enrichment of total Fe and reactive Fe which have been linked to carbon loss [48]. Additionally, Kunarso *et al*. reported that Fe concentration in peatlands was highest in peatland converted to a plantation with perennial plant cover [52]. Moreover, a recent study in tropical forests showed that Fe promotes soil OM decomposition under limited oxygen [53]. Similarly, Fe-rich fens also showed increased C-GHG production via Fe reduction coupled with OM decomposition under oxygen-limited conditions [54]. Thus, it is intriguing to investigate the underestimated Fe-cycling pathways and mechanisms behind its role in the carbon loss in both managed and natural peatlands. Tropical peatlands possess a markedly different chemistry with low pH, higher aromaticity, and elevated temperatures, resulting in increased demands for high-energy TEAs such as Fe, resulting in it becoming the dominant pathway for OM [55].

**Figure 1:**
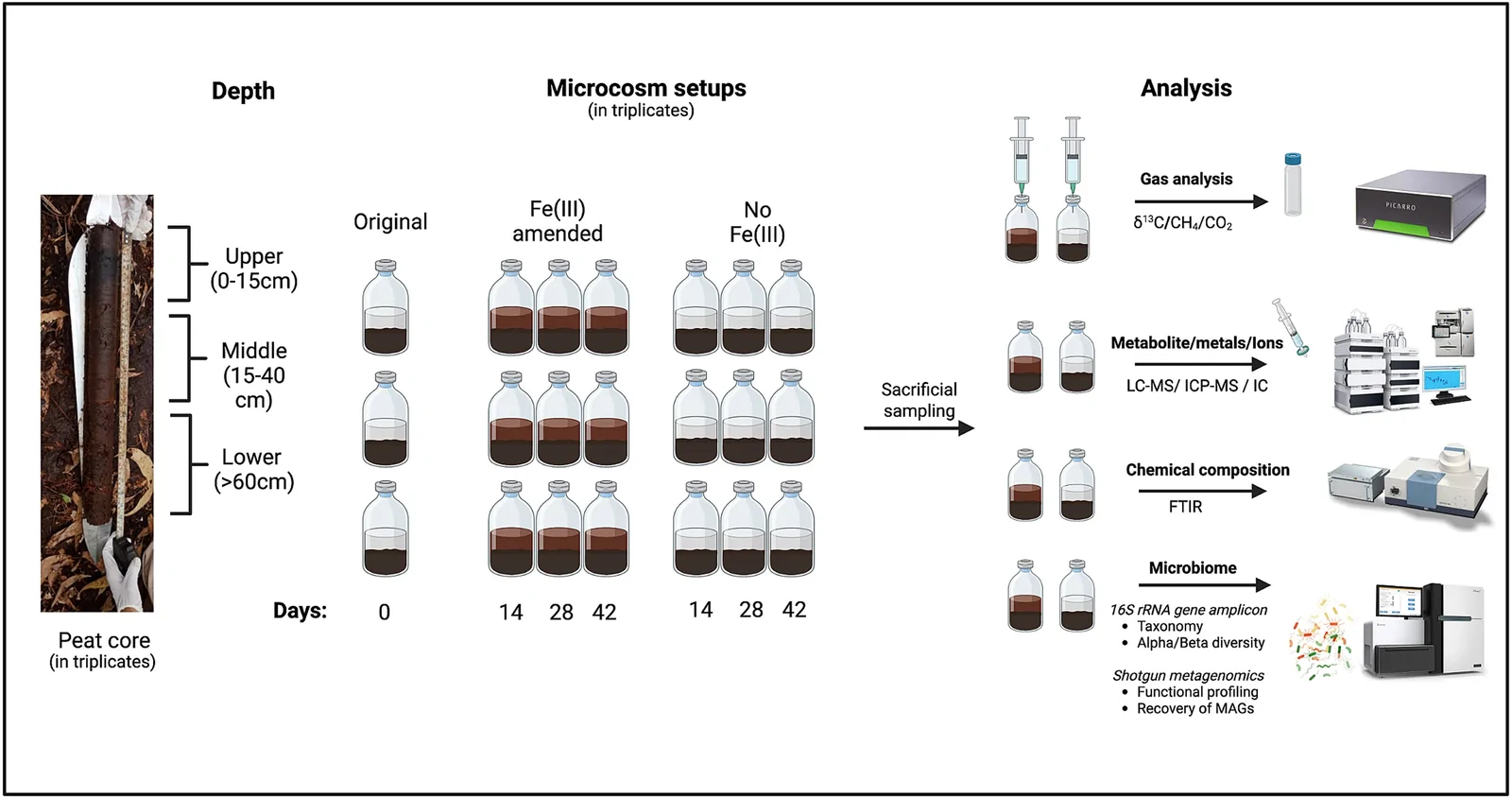
Schematic representation outlining the sequential steps, timeline, and experimental setup of the study.

This study aims to assess the role of Fe(III) in (i) C-GHG production from peat and (ii) OM degradation and transformation. To achieve these aims, we conducted an Fe(III)-amended microcosm experiment with peat from three different depths collected from a fiber crop plantation (Riau province, Sumatra, Indonesia), as shown in Fig. 1. We measured C-GHG production in response to the Fe(III) amendment and observed the shifts in the microbial community composition and function over time using amplicon and shotgun metagenome approaches. To obtain a deeper insight into OM degradation resulting from Fe(III) amendment, we monitored the changes in OM transformation by studying metabolite profiles and functional groups using liquid chromatography–mass spectrometry (LC–MS) and Fourier transform infrared spectroscopy (FTIR), respectively. Additionally, we studied isotopic signatures of C-GHG to understand the origin of the carbon pool.

## Materials and methods

### Site description and peat properties

To understand the mechanistic pathways related to Fe(III) and C-GHG production, the peat samples for the study were collected from a 4-year old *Acacia crassicarpa* fiber crop plantation in Riau Province, Sumatra, Indonesia. These plantations were dedicated to water table manipulation experiments at coordinates 0.516545° N and 102.107758° E as described in Taillardat *et al*. [56]. The peat sampling was performed in November 2022 by considering the September to November window of the wettest months (>300 mm month^−1^). During sampling, three peat cores of 1 m were collected in the area between trees to avoid roots, using a Russian corer. Cores were segregated into three depths: upper (0–15 cm), middle (15–40 cm), and lower (>60 cm) depths where the water table depth of the plantation plot was maintained at −40 cm. Cores from the same depths were mixed thoroughly and composite samples were prepared for each depth. Physicochemical parameters such as dissolved oxygen (DO), electrical conductivity (EC), pH, temperature, and moisture were measured from these three different peat depths using a HANNA multiparameter probe (HI98194). Peat pH was measured using a Sartorius pH meter PB-10 model.

### Peat microcosms

Peat from three different depths, i.e. upper: 0–15 cm, middle: 15–40 cm, and lower: >60 cm, was used for microcosm establishment (Fig. 1). Fifteen grams of peat was added with 45 ml of filter sterilized autoclaved distilled water into a 100 ml glass serum vial. An amount of 5 mM Fe(III) (FeCl_3_) was added as TEA in Fe-amended setups. Similarly, an unamended setup was prepared without Fe. The N_2_ gas was not purged in the headspace to retain peat characteristics from the field conditions, as much as possible. Samples were prepared in triplicates and were sacrificially sampled at Day 0, 14, 28, and 42. Gas from headspace was withdrawn at each of these timepoints for estimation of CO_2_ and CH_4_ generation using Picarro Gas analyser (G2201-i, Picarro Inc., USA) (see detailed supplementary materials and methods). Slurry samples were recovered from all four timepoints for DNA extraction (see detailed supplementary materials and methods). To study the chemical composition, metabolite profiling and FTIR analysis were performed on Day 0 and last day slurry samples, as shown in Fig. 1 (details provided in the supplementary materials and methods). Amplicon and shotgun sequencing were performed to assess the change in taxonomic and functional profile during the incubation setup (details provided in the supplementary materials and methods). DO and ORP readings were not taken during the sacrificial sampling as the study was setup in a laboratory close to the sampling site with no access to an anaerobic chamber.

## Results and discussion

### Geochemical nature of the peat

The detailed description of the geochemistry of the peat samples is summarized in Table 1. In brief, the peat samples from the three depths (0–15 cm: upper, 15–40 cm: middle, and >60 cm: lower) were highly acidic in nature with pH ranging from 2.70 to 2.76. No significant variations were observed for some of the tested on-site physicochemical parameters, such as DO, and temperature across the three depths. However, EC was negatively correlated with depth (*r* = −0.824, *P* = .006), while moisture content showed a positive correlation with depth (*r* = 0.691, *P* = .03). The concentration of major anions such as sulfate (*r* = 0.82, *P* = .006) and phosphate (*r* = 0.62, *P* = .07) increased with increasing depth. However, no significant (*P* > .05) variation was observed for nitrate and nitrite concentrations across the depth. In addition, concentrations of Fe (*r* = −0.79, *P* = .011) and Cu (*r* = −0.82, *P* = .006) decreased with depth while other metals, such as Zn, Ni, As, and Mn did not vary with depth. The pH of the microcosm setup was collected at the end of the sacrificial sampling. A pH drop of 0.5 unit was noted in all Fe(III)-amended setups as shown in Supplementary Table SS1. The pH remained steady throughout the incubation. The limitations of using FeCl_3_ therefore constitute a multifactor perturbation with pH and/or chloride ions playing a potential role in the observed changes in CO_2_ and CH_4_ production in amended setups.

**Table 1:** Geochemical profile of the peat cores showing three different depths, i.e. upper 0–15 cm, middle 15–40 cm, and lower depth >60 cm.

Parameters	Upper depth	Middle depth	Lower depth	Correlation with depth (r)	*P* value
Depth (cm)	0–15	15–40​	>60​	​	​
EC (μS/cm)	104.33 ± 17.04	90.00 ± 8.19​	67.33 ± 10.41​	−0.824​	.006​
Moisture (%)	73.71 ± 8.77	76.71 ± 10.30​	89.60 ± 0.64​	0.691	.039​
DO (%)	0.73 ± 0.06	0.70 ± 0.06​	0.70 ± 0.03​	−0.433​	.244
pH	2.70 ± 0.14	2.71 ± 0.10​	2.76 ± 0.14​	0.219	.571​
Temp (°C)	30.89 ± 0.51	30.68 ± 0.41​	30.53 ± 0.38​	−0.386	.304​
Sulfate (mg/kg)	BDL	0.77 ± 0.01​	2.31 ± 0.02​	0.821	.006​
Nitrate (mg/kg)	0.87 ± 0.76	0.65 ± 0.49​	0.96 ± 1.01​	0.057	.883​
Nitrite (mg/kg)	26.59 ± 17.83	30.05 ± 6.64​	30.98 ± 17.77​	0.144​	.711​
Phosphate (mg/kg)	4.12 ± 0.01​	4.32 ± 0.35​	4.701 ± 0.53​	0.620	.074​
Fluoride (mg/kg)	0.56 ± 0.76​	4.72 ± 1.46​	2.45 ± 0.37​	0.410	.272​
Chloride (mg/kg)	2.16 ± 1.76​	2.32 ± 0.83​	4.24 ± 2.92​	0.446	.228​
Bromide (mg/kg)	4.47 ± 2.25​	3.33 ± 0.95​	2.67 ± 0.34​	−0.532​	.139​
Na (μg/kg)	1701.87 ± 58.47​	2104.72 ± 96.43​	1916.47 ± 2.97​	−0.600	.0871​
Mg (μg/kg)	796.14 ± 4.30​	1180.55 ± 107.76​	1058.54 ± 26.99​	−0.599	.088
Ca (μg/kg)	185.48 ± 17.22​	166.21 ± 18.69​	93.32 ± 10.66​	−0.676	.084
Cr (μg/kg)	12.39 ± 0.60​	9.41 ± 0.27​	11.2 ± 1.91​	−0.143​	.712
Mn (μg/kg)	21.79 ± 4.51​	11.01 ± 0.57​	17.15 ± 2.86​	−0.215​	.577
Fe (μg/kg)	819.83 ± 4.08​	702.27 ± 48.81​	564.05 ± 29.25​	−0.790	.011​
Ni (μg/kg)	7.52 ± 0.21​	6.59 ± 0.77​	6.46 ± 0.8​	−0.245	.523
Cu (μg/kg)	4.80 ± 0.26​	3.37 ± 0.44​	2.10 ± 0.15​	−0.825	.006
Zn (μg/kg​)	7.49 ± 1.27​	4.78 ± 0.76​	4.08 ± 1.29​	−0.442	.233​
As (μg/kg)	3.28 ± 0.74​	8.95 ± 4.82​	11.82 ± 3.83​	0.368​	.328

Note: BDL, below detection limit.

### Carbon greenhouse gases (CH_4_ and CO_2_) production

**Figure 2:**
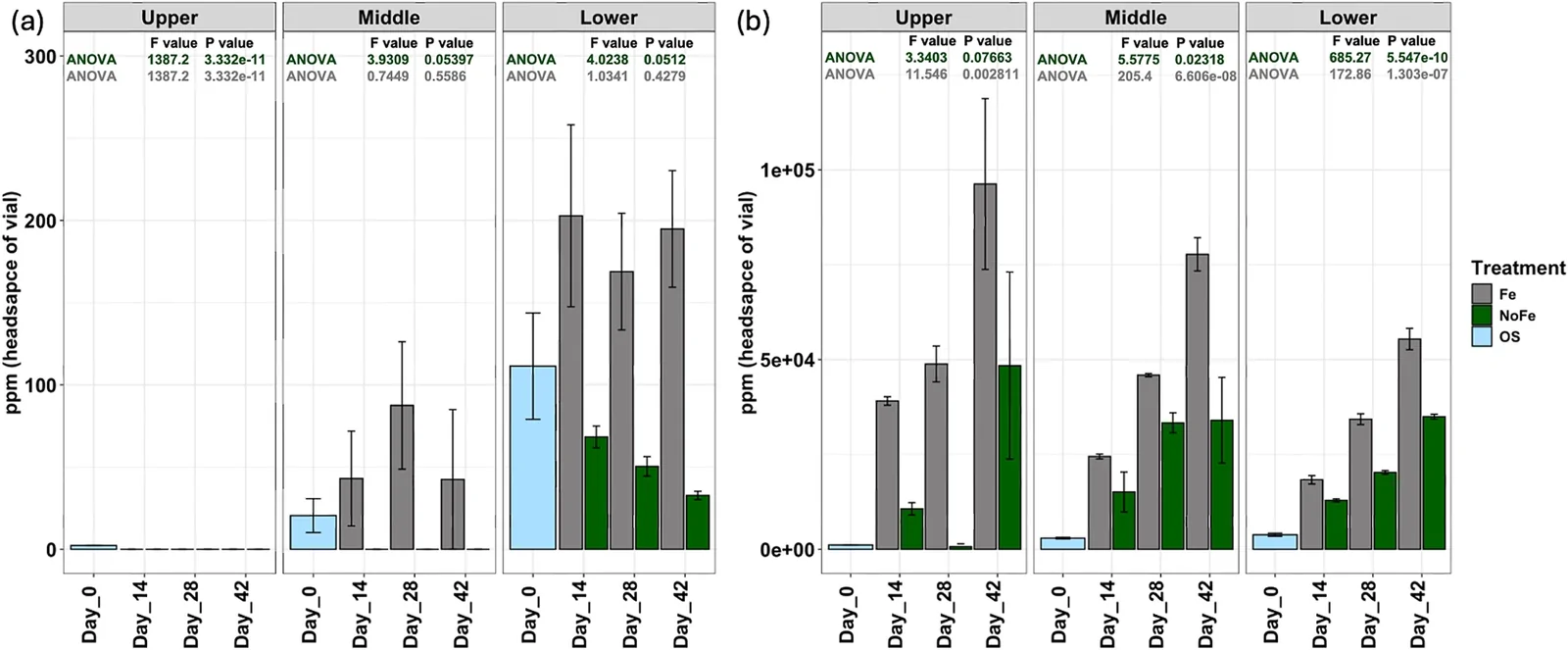
Bar graph representing C-GHG production during incubation. CH_4_ (a) and CO_2_ (b) production profiles from the upper, middle, and lower depths of the peat microcosm setup. OS, Day 0 samples; NoFe, unamended, Fe-amended with Fe. Triplicates are technical replicates derived from the composite samples.

The C-GHG production in response to Fe(III) amendment across the different depths was quantified to assess the dynamics of CH_4_ and CO_2_ production over 6 weeks (Fig. 2). At Day 0 of incubation, CH_4_ amount was higher in the lower depth microcosms (111 ± 56 ppm) than middle (30.7 ± 3.32 ppm) and upper (2.3 ± 0.1072 ppm) ones (Fig. 2a). In unamended setups, CH_4_ production was undetectable in the upper and middle depths while a gradual decline in CH_4_ (*P* = .051) was observed in lower depth. In the Fe-amended setups, CH_4_ production was gradually increased with a maximum production at 28 days in the middle depth. On the contrary, CH_4_ was rapidly produced by Day 14 and remained constant throughout the experiment (*P* = .4279) in the lower depth.

The CO_2_ production levels were severalfold higher than CH_4_ production. In contrast to the CH_4_ production, setups amended with Fe(III) had increased CO_2_ production compared to the respective unamended controls (Fig. 2b). This trend was similar across all the depths. At 42 days, the upper depth amended with Fe(III) had the highest CO_2_ concentration (96 259 ± 38 995 ppm) compared to the middle (77 726 ± 7 588 ppm) and lower (55 398 ± 4 877 ppm) depths. The decrease in CO_2_ production with depth is consistent with an earlier report by Moore and Dalva [29]. In contrast, CH_4_ production increased with depth. CO_2_ production in unamended control followed a similar trend to Fe(III) amendment; however, the total CO_2_ generation in the control was nearly half across all time points and depths. Overall, CH_4_ and CO_2_ production between treatments, timepoints, and depth were found to be significantly different (Supplementary Tables SS2 and SS3).

Our results suggested that the presence of Fe(III) stimulates the OM decomposition as evidenced by an increased CO_2_ concentration across the three depths under controlled experimental conditions. The Fe(III) is one of the most thermodynamically favorable TEAs under oxygen-limiting conditions; however, the wide difference in the CH_4_ and CO_2_ production detected between treatments suggests substrate competition between Fe(III) reducers and methanogens [57, 58]. Furthermore, this difference in scale of CH_4_ and CO_2_ production is consistent with field observations in the tropics [56, 59–61]. Interestingly, a similar pattern was observed in a previous study by Emsens *et al*., which reported that Fe-rich fens had a positive influence on C-GHG production via Fe reduction coupled with OM decomposition under oxygen-limited conditions [54]. In the microcosm setups here, easily metabolizable carbon in the upper depth likely supports more fermentative and/or microaerophilic respiration processes resulting in higher CO_2_ production compared to the middle and lower depths where possibly more recalcitrant compounds resistant to degradation are more abundant [62]. This suggests that the microbial composition and metabolic potential vary across peat depth though abiotic transformations may also contribute to CO_2_ production across depths. A key limitation of this study was the absence of ORP measurement through the incubation as the redox state influenced both the Fe and CH_4_ cycling. Notwithstanding this limitation, the trends observed in CH_4_ and CO_2_ production across depths and treatments provide valuable insights into the potential interactions between Fe(III) availability and C-GHG dynamics.

### Isotopic signature of detected CO_2_

We next asked the following questions related to the origin of the carbon produced as C-GHG: (i) does the C-GHG originate from a more labile or recalcitrant carbon pool and (ii) how does peat depth affect the origin of the C-GHG? To address these questions, we adopted a δ^13^C-CO_2_ isotopic signature approach. Our results revealed that the δ^13^C-CO_2_ isotopic signature values were similar for upper: −22 ± 0.45%, middle: −22.72 ± 1.8%, and lower: −22.86 ± 1.45% depths at Day 0 (Supplementary Table SS4). Interestingly, substantial changes in the isotopic signature values over time were observed in the microcosm setups. At 42 days, the δ^13^C-CO_2_ isotopic signatures of unamended setups were − 27.6 ± 0.01%, −28.6 ± 1.66%, −28.3 ± 0.12% for upper, middle, and lower depths, respectively, while the δ^13^C-CO_2_ isotopic signatures of Fe(III)-amended setups were −28.6 ± 0.34%, −29.4 ± 0.33%, −30.1 ± 0.15% (Supplementary Table SS4) in the upper, middle, and lower depths, respectively.

Temporal variations in δ^13^C-CO_2_ over the incubation period were primarily due to the changes in the microbial community composition and the preferential utilization of δ^13^C substrates present in the peat [63]. The decrease in δ^13^C-CO_2_ value in the lower depth upon Fe amendment suggested the decomposition of recalcitrant compounds. It has been reported that substrates with higher δ^13^C including sugars and organic acids decomposed much faster than recalcitrant compounds like lignin or aromatic compounds [64]. Lignin and aromatic compounds are typically depleted in δ^13^C compared to bulk plant materials [65]; consequently lower δ^13^C-CO_2_ values were recorded in the deeper depth as a result of their degradation. Our results suggest that Fe(III) amendment supports the production of CO_2_ from more recalcitrant sources of OM in the lower depths. The upper and middle depths have higher production of overall CO_2_ with higher δ^13^C-CO_2_ suggesting that more labile or easily metabolizable OM was converted to CO_2_.

To complement the initial findings on the origin of C-GHG, we next considered δ^13^C-CH_4_ to determine whether the methanogenesis is occurring via acetoclastic and/or hydrogenotrophic pathways. The δ^13^C-CH_4_ isotopic signatures of CH_4_ were analyzed only from the lower depth, as CH_4_ was undetectable in the upper depths. By the end of the incubation, δ^13^C-CH_4_ values were more enriched in the control setup (−37.7 ± 3.9%) than the Fe(III)-amended setup (−63.7 ± 4.8%) (Supplementary Fig. SS1). Thus, these δ^13^C values suggest the dominance of acetoclastic methanogenesis [56]; however, the more positive δ^13^C-CH_4_ value in the unamended setups supports methane oxidation, i.e. CH_4_ to CO_2_ [28], which corresponds to the decrease in CH_4_ production as depicted in Fig. 1.

### Influence of Fe(III) on organic matter transformation

**Figure 3:**
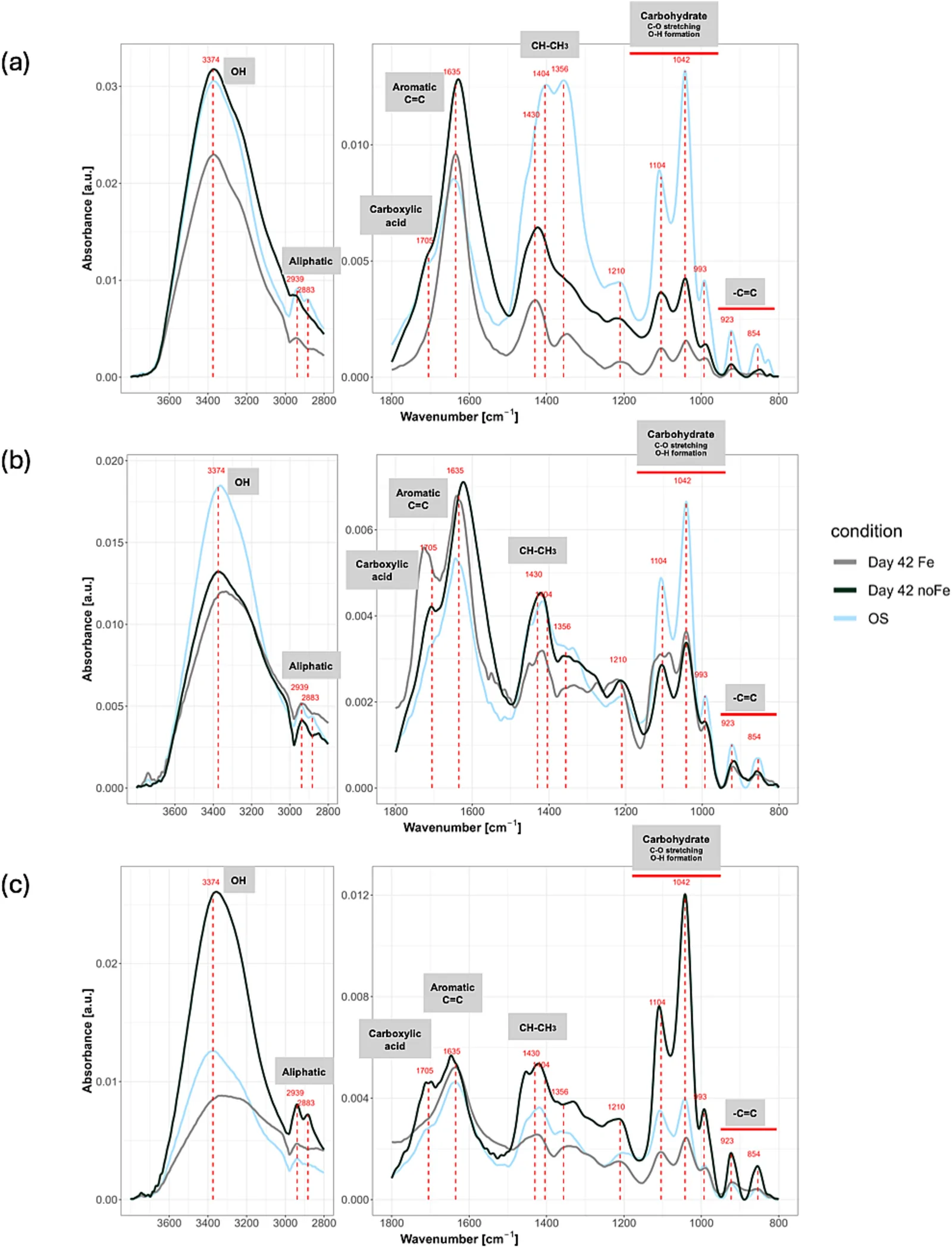
A three-panel figure showing average FTIR spectra of peat samples across different depths and treatments. (a) Upper, (b) middle, and (c) lower depth peat samples under different treatments. Light blue indicates Day 0 samples (OS), gray indicates Fe(III)-amended peat setup at Day 42, and dark green indicates unamended peat (NoFe) setup at Day 42.

Having established that C-GHG originated from different peat OM in a depth-dependent manner, we next investigated the chemical groups that contribute to the production. To achieve this, we conducted Attenuated Total Reflectance–Fourier Transform Infrared spectroscopy (ATR-FTIR) analysis at the Infrared Spectro/Microscopy (ISMI) beamline of the Singapore Synchrotron Light Source, enabling high-resolution profiling of chemical groups across different depths and treatment conditions. The spectral vibrations of the relevant chemical compounds are presented in Fig. 3. At Day 0, depth-dependent spectral variations were observed. Vibrational peaks associated with C–O of carbohydrate moieties (1104–1042 cm^−1^) were more prominent in the upper and middle depth, whereas CH–CH_3_ stretching (1404–1356 cm^−1^) was more prominent in the upper depth. Higher intensity of carbohydrate peak in upper depth as compared to lower depth indicates that it is less decomposed due to it receiving fresh plant leaf litter rich in carbohydrate compounds on a continuous basis [66]. Furthermore, this spectral variation could be explained likely by the differential rate of deposition of plant litter of the overlying vegetation, shift in hydrology, degree of decomposition of OM, mineralogy, microbial activities, and environmental fluctuations across different depths over the years [67, 68].

During the incubation study, a prominent shift in the absorbance spectra was observed across all the depth and treatments (Fig. 3) and the significance between the intensities of these major peaks has been represented in Supplementary Fig. SS2. The Fe(III) amendment in the upper depth led to the reduction in intensity of the bands between 1104 and 1042 cm^−1^ (stretching vibration of C–O of carbohydrate), 1404 and 1356 cm^−1^ (CH–CH_3_ stretching), and 2939 and 2883 cm^−1^ (aliphatic stretching) compared to the control setups (Fig. 3a). In the middle depth, aromatic C=C stretching (absorbance peak at 1635 cm^−1^) was more pronounced in both Fe and non-Fe-amended setups. On the other hand, carboxylic acid groups (1705 cm^−1^) were enriched only in the Fe(III)-amended setups (Fig. 3b). These results suggested that a reduction in the carbohydrate component of the peat in both the depths was indicative of decomposition of such compounds by the enriched microbial members and/or abiotic processes, whereas incremental changes in carboxylic acid and aromatic groups in the upper and middle depths were indicative of higher degree of oxidation and decomposition processes that led to the generation of such functional groups [66]. Based on the changes in ATR-FTIR spectra band intensities, we propose that Fe(III) amendment stimulated the decomposition process of peat. Reduction in the carbohydrate compounds as detected in the ATR-FTIR spectra, further confirmed that the enriched microbial members in the Fe(III)-amended setups preferred easily fermentable carbohydrates and released higher CO_2_ than non-amended setup as shown in Fig. 2b. In comparison to the above peat depths, the Day 0 lower depth sample showed quite lower spectral bands for carbohydrate, aromatic, and carboxylic acid moieties (Fig. 3c). However, upon incubation, Fe(III)-amended and unamended setups showed different decomposition behavior indicating that microbial communities enriched in these two setups preferred different substrates for utilization. Overall, these differences in peat OM composition upon amendment might be used as a proxy for decomposition potential and reflect changes in CH_4_ and CO_2_ production.

Additionally, we investigated the metabolite profile of the peat samples by LC–MS based metabolomics. Among the identified classes of metabolites, carbonyl compounds, amino acids, peptides and analogs, amides, lipids, steroidal glycosides, phosphates, and ketals constituted the major groups of the peat metabolome at Day 0, which varied with different depths (Supplementary Fig. SS3a–c). Our results revealed that at the end of the incubation, the metabolite profiles in the lower depth showed significant changes in amino acids, peptides, and analogs (*P* = .036) in the Fe(III)-amended setups.

A slight increase in the carboxylic acid content in the upper and middle depths was corroborated with the ATR-FTIR data under Fe(III) amendment. However, in the upper and middle depths, a decrease in amino acids, peptides, and analogs, as well as lipids and steroidal glycosides, was observed. Contrastingly, in the lower depth carbohydrates and derivatives showed a decrease in the relative abundance upon Fe(III) amendment. This suggests that the enriched microbial community metabolized these compounds and derived C and N from them for their metabolism, which ultimately led to the observed increase in C-GHG production (Fig. 2). Furthermore, the increase in amino sugar content reflects an increase of microbial activities across each depth, as plants are not known to produce them [69, 70]. These findings further support that Fe promotes microbial metabolism and CO_2_ production through Fe(III)-mediated decomposition of organic compounds.

### Niche-specific responses of microbial communities to Fe(III) supplementation

**Figure 4:**
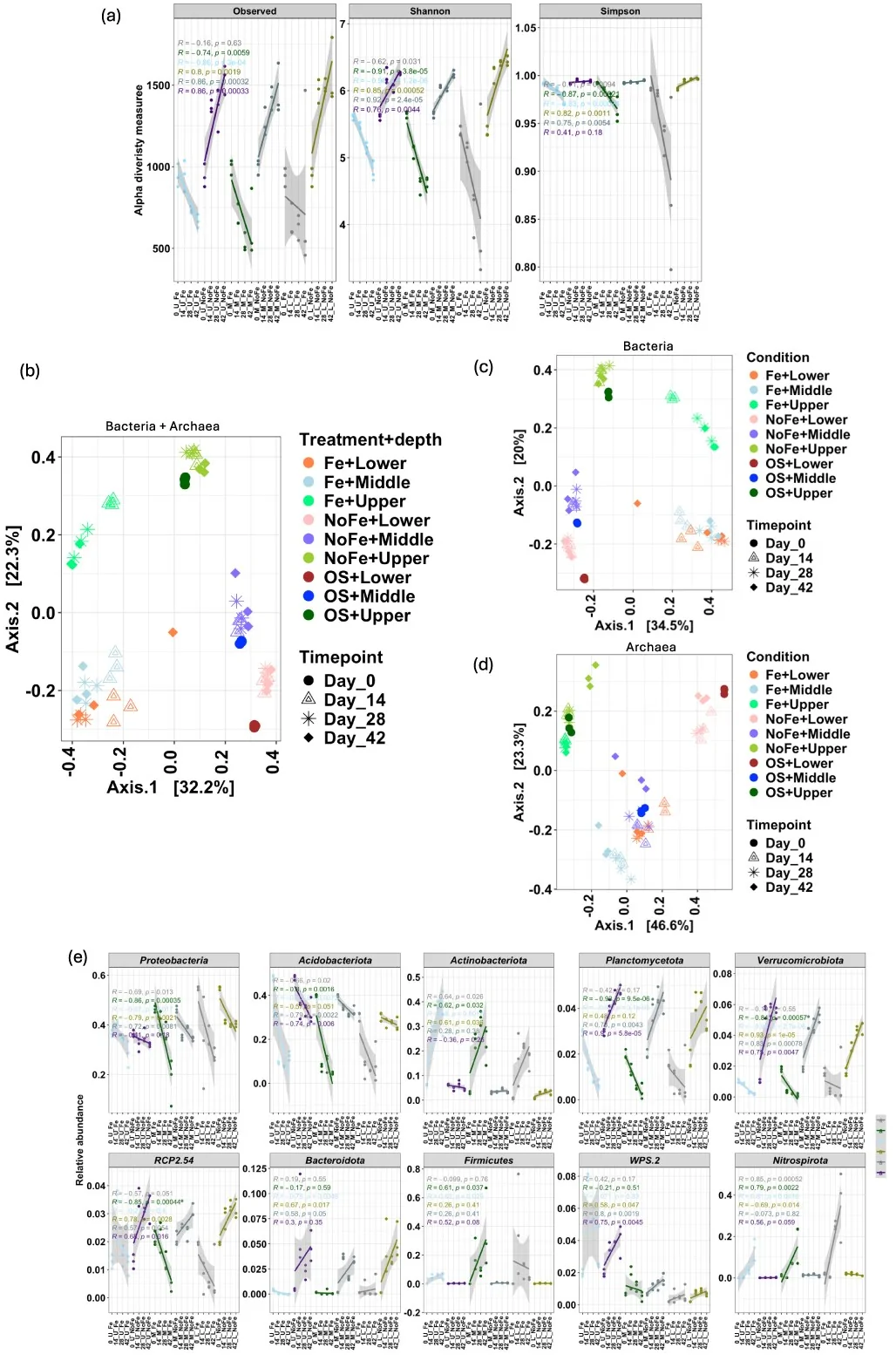
A five-panel figure showing changes in alpha and beta diversity of microbial communities across treatments, depths, and timepoints in peat samples. (a) Treatment- and time-dependent changes in the alpha diversity of the microbial community. (b) Changes in the overall microbial community composition over time and treatment. The three-way interaction between Condition, Depth, and Timepoint is significant (PERMANOVA: *R*^2^ = 0.02536, *F* = 2.5048, *P* = .0001). The two-way interaction between condition and depth is significant (PERMANOVA: *R*^2^ = 0.14245, *F* = 8.45, *P* = .0001), followed by Timepoint and Condition (PERMANOVA: *R*^2^ = 0.03331, *F* = 1.5943, *P* = .0883). (c) Changes in the bacterial community composition over time and treatment. The three-way interaction between Condition, Depth, and Timepoint is significant (PERMANOVA: *R*^2^ = 0.03138, *F* = 1.6129, *P* = .0315); the two-way interaction between Condition and Depth is significant (PERMANOVA: *R*^2^ = 0.11909, *F* = 4.9998, *P* = .0001). (d) Changes in the archaeal community composition over time and treatment. The three-way interaction between Condition, Depth, and Timepoint is significant (PERMANOVA: *R*^2^ = 0.01629, *F* = 2.2257, *P* = .0117). The two-way interaction between Condition and Depth is significant (PERMANOVA: *R*^2^ = 0.12865, *F* = 10.271, *P* = .0001), followed by Timepoint and Depth (PERMANOVA: *R*^2^ = 0.07424, *F* = 1.9625, *P* = .0115). (e) Abundance of key bacterial phyla across the different depths and treatments. Correlation values are presented in the respective figures. OS, original sample; Fe, amended with Fe(III); NoFe, unamended setup; U, upper; M, middle; L, lower; and numbers denote the days.

To understand the influence of Fe amendment on the microbial community composition in a depth-dependent manner, we performed *16S rRNA* gene-based amplicon sequencing for all the sampling timepoints. Day 0 samples from three different depths were considered as baseline data. Our amplicon data revealed a prominent shift in species richness and evenness in both amended and unamended setups compared to Day 0. The number of observed species in terms of amplicon sequence variant (ASVs), richness, and evenness decreased in Fe-amended setups over time, while unamended setups reflected an opposite trend (Fig. 4a). The decreased diversity in the Fe amended than unamended setups indicated that experimental conditions enriched specific microbes due to the selection pressure of Fe. Our results are in line with a previous study, where an iron oxide ferrihydrite showed decrease in species richness [71]. Beta diversity analysis confirmed statistically significant (permutational multivariate analysis of variance: PERMANOVA *F* = 15.706, *P* = 1e-04 ***) changes in the total microbial composition of the Fe-amended setups compared to unamended controls (Fig. 4b). Furthermore, PERMANOVA showed significant difference bacteria (PERMANOVA *F* = 17.857, *P* = 1e-04 ***) and archaeal (PERMANOVA *F* = 6.0817, *P* = 1e-04 ***) communities upon Fe amendment (Fig. 4c and d). Strikingly, the addition of extraneous Fe resulted in the enrichment of a similar community structure overtime in the middle and lower depths as compared to upper depth. However, the microbial profile of unamended setups did not show much variation from Day 0 across depths, highlighting the influence of Fe on shaping the microbial community composition.

The bacterial community composition revealed that the abundance of *Acidobacteriota* and *Proteobacteria* significantly decreased over time across the three depths, with the maximal reduction noted in the Fe-amendment setups (Fig. 4c). Interestingly, a few microbial groups showed contrasting trends. For instance, members belonging to *Verrucomicrobiota*, *Planctomycetota*, *Bacteriodota*, and RCP2.54 decreased in Fe-amended setups over time, and vice versa for unamended setups (Supplementary Table SS5). However, the relative abundance of *Actinobacteriota*, *Nitrospirota*, and *Firmicutes* populations enhanced upon Fe addition (Fig. 4c).

**Figure 5:**
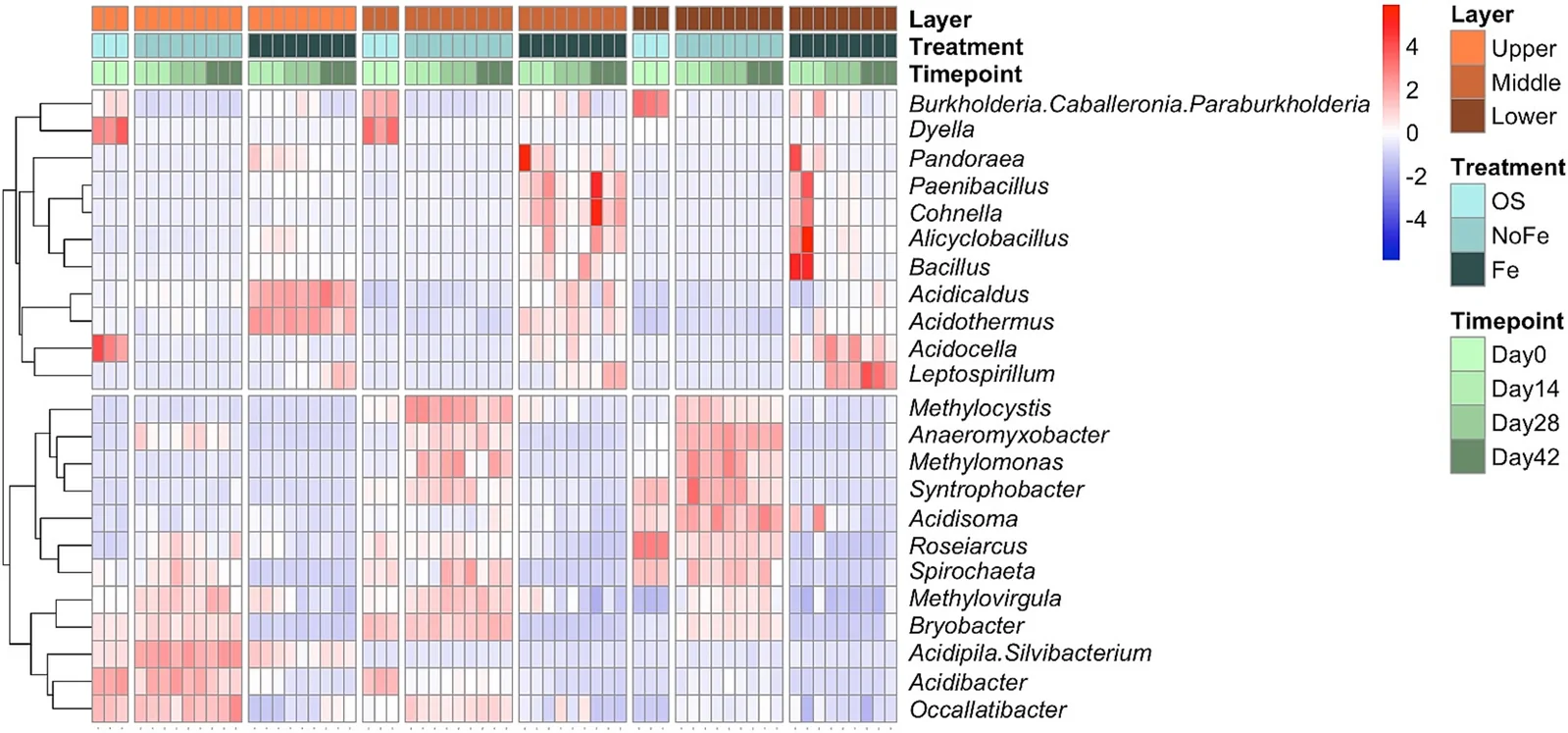
A heatmap displaying the relative abundance of major bacterial taxa known to be involved in Fe metabolism, methanotrophy, and carbon metabolism.

A more pronounced shift was observed at the genus level (Fig. 5). Several Fe-reducing, methanotrophic, and organic carbon-degrading microbes showed prominent changes in their abundance patterns over time. Among the known Fe reducers, *Alicyclobacillus* and *Acidicaldus* showed a continuous increase in their relative abundance across the three depths in Fe(III)-amended setups, while *Acidocella* increased maximally in the middle and lower depths [72–74]. A pairwise *post hoc* comparison using Dunn’s test showed statistically significant (*P.*adj <.05) changes in the relative abundance of these genera across treatments and depths (Supplementary Data SS1). These dissimilatory Fe reducers mineralize peat organic carbon coupled to Fe reduction for their growth and metabolism, thus underlying their significance in both carbon and Fe cycling.

Interestingly, *Leptospirillum*, a known acidophilic Fe(II) oxidizer, was substantially enriched (*P*.adj <.05, *post hoc* using Dunn’s test) in the Fe-amended setup across lower and middle depths, likely by utilizing Fe(II) generated by the Fe(III)-reducing microbes [75]. *Leptospirillum* has been previously reported to be abundant in peat swamp forests due to its ability to live in strongly acidic environments and support Fe cycling [76]. This cyclical oxidation and reduction of Fe is reflected in the dynamic changes in the abundance of Fe(III) reducers and oxidizers. On the contrary, control setups showed an interesting increasing trend of methanotrophic/methylotrophic (*Methylovirgula*, *Methylocystis*, and *Methylomonas*) members. However, only *Methylovirgula* showed a significant (*P* < .05) increase with time across three depths in unamended setups (Supplementary Fig. SS6) whereas *Methylocystis* and *Methylomonas* showed a significant (*P* < .05) increment in upper and middle depths, respectively. The increment of these members confirmed the consumption of CH_4_ produced during incubation in the lower depth, as reflected in Fig. 2. These methanotrophs have been previously reported to consume nearly 30%–40% of the CH_4_ produced naturally in peat or wetlands [77]. Moreover, among the detected organic carbon degraders prominent increase of *Acidothermus* was seen in Fe-amended setups. Higher abundance of this genus in the upper depth corroborated with the higher CO_2_ production indicating its role in decomposition of peat OM. *Acidothermus* was previously reported to perform cellulolysis in drained peatland sites [78, 79]. Similarly, *Paenibacillus*, *Bacillus, Pandoraea*, and *Cohnella* were enriched in Fe-amended setups at specific depths (*P*.adj <.05 *post hoc* using Dunn’s test). These members have been previously reported to perform Fe reduction in diverse environments including paddy soils and acid mine drainage [74, 79–81]. In the present study, the enrichment of such members reveals that along with fermentative metabolism these members can perform Fe reduction upon the availability of Fe.

On the contrary, the control setup showed enrichment of distinct organic carbon degraders including *Spirochaeta*, *Syntrophobacter, Bryobacter, Occallatibacter*, and *Roseiarcus.* These members drive the degradation of plant-derived carbon polymers, sugars, and proteinaceous substances in peatlands and wetlands [78, 82–85]. Hence, production of CO_2_ in control setup is due to their fermentative metabolism that produces intermediary substrates, which are eventually utilized by secondary fermenters to produce CO_2_. This corroborates with the production of CO_2_ as shown in Fig. 2b.

Furthermore, we investigated the changes in archaeal populations over time, as peat is known to harbor archaeal populations that play an important role in carbon and metal cycling [86, 87]. Archaeal reads were selected from the V4-V5 region of the *16S rRNA* gene and analyzed separately. *Thermoplasmatota* and *Crenarchaeota* were the predominant archaeal phyla detected (Supplementary Fig. SS4). *Thermoplasmatota* members increased over time with Fe amendment across the different depths, while the opposite trend was observed for *Crenarchaeota*. *Thermoplasmata* and *Nitrososphaeria* constituted the major classes of the archaeal domain in middle and lower depths whereas, *Bathyarchaeia* was dominant in the lower depth at Day 0 (Supplementary Fig. SS5). However, *Thermoplasmata* members increased with Fe amendment, while *Nitrosophaeria* and *Bathyarchaeia* decreased over time. In addition, the known hydrogenotrophic, acetoclastic, and methylotrophic methanogens (*Methanosarcinia, Methanomicrobia, Methanobacteria*, and *Methanomethylicia*) were found only in the middle and lower depths accounting for ~0.1% and ~0.2%, respectively, at Day 0. These depths are waterlogged and thus support the growth of these anaerobic microorganisms in natural conditions and release CH_4_ via methanogenesis. *Methanobacteria* was the only class of methanogen that increased in the Fe(III)-amended setups in middle and lower depths as compared to their counterpart. However, the rest of the methanogens, i.e. *Methanomicrobia* and *Methanosarcinia* showed higher enrichment in the middle and lower depths of the control conditions. The reduced enrichment of methanogenic members in the Fe-amended setup was corroborated with the minimal generation of CH_4_ compared to the generation of CO_2_. This suggests that the bacterial population including Fe reducers may have outcompeted the methanogenic archaeal population. In addition, the Fe-rich environment may suppress methanogenesis due to competitive exclusion between dissimilatory Fe reducers and methanogenic archaea, competing for common electron donors [88–92]. Moreover, the increase in CH_4_ production may not reflect the change in abundances of methanogens. Similarly, Wilson et al. observed CH_4_ increase during warming, with no increase in relative abundance of methanogens [93].

### Metagenomic profiling of organic carbon degrading and biogeochemical pathways via gene-centric approach

**Figure 6:**
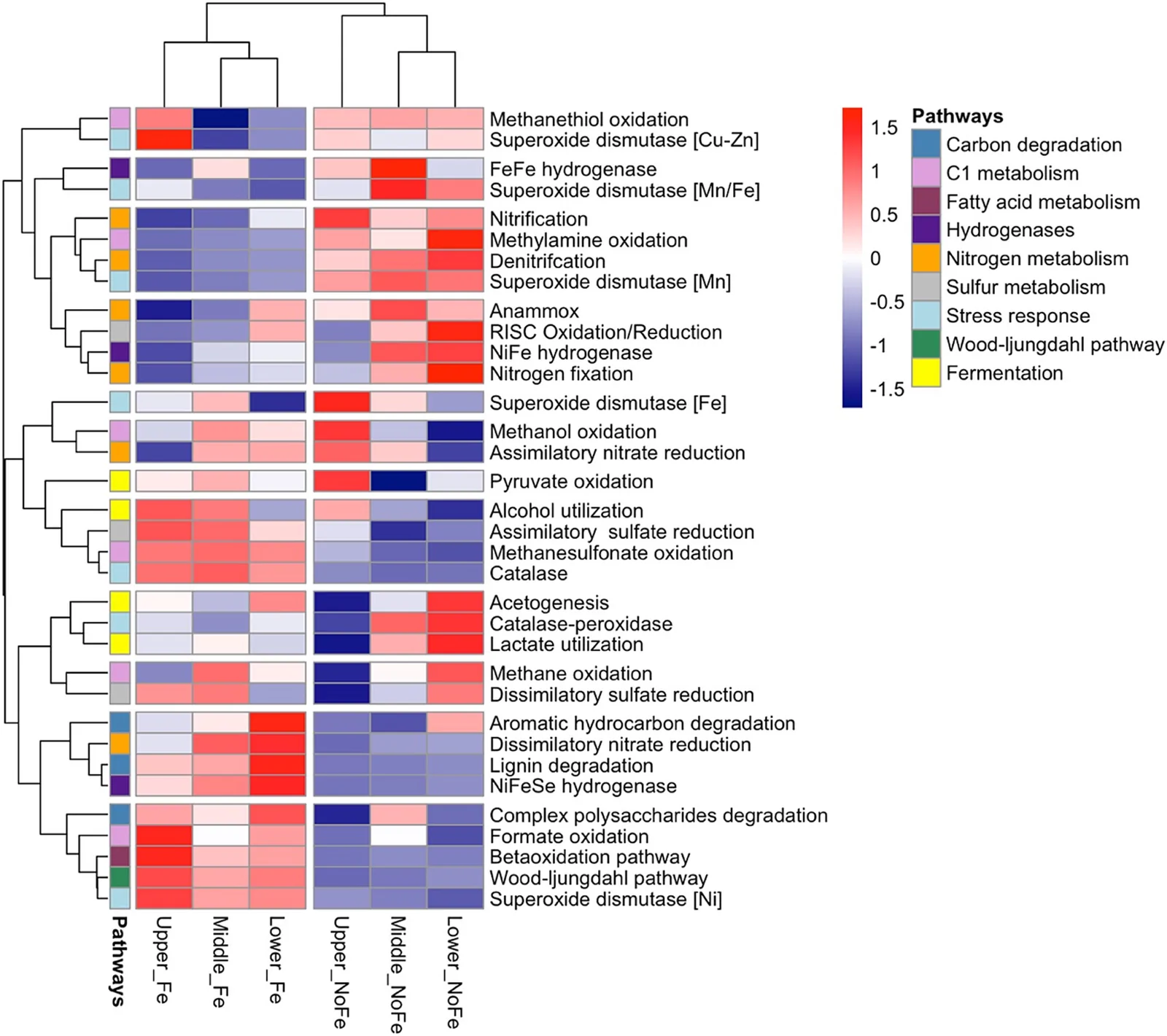
A figure presenting a gene-centric analysis of key metabolic pathways involved in the degradation of complex organic carbon compounds along with sulfur, nitrogen and stress response metabolisms in peat samples.

Plant polymers, phenolic compounds, and hydrocarbons are the key carbon compounds that get degraded in the peat system [94]. We screened the metagenomic functional potential of the peat microbial community to decipher the indigenous potential of the microbes involved in this process. Our functional profile data suggest that Fe amendment enhanced the degradation of polysaccharides, lignins, tannins, polyphenols, and aromatic hydrocarbons as depicted in Fig. 6. However, lower depth showed a substantial increase in the abundance of these complex organic carbon degradation genes. Additionally, beta-oxidation pathway and Wood–Ljungdahl pathway genes were also enriched in Fe-amended setups. In order to further delineate which genes were actually differentially abundant among these metabolic categories as depicted in the heatmap (Fig. 6); we performed MaAsLin2 and found out that genes involved in lignin degradation (*protocatechuate 3*,*4*-*dioxygenase, protocatechuate 4*,*5*-*dioxygenase,* vanillate/3-*O*-methylgallate *O*-demethylase), aromatic carbon degradation (toluene-4-monooxygenase system, hydroxylase component subunit alpha, toluene-4-monooxygenase system, hydroxylase component subunit beta, naphthalene 1, 2-dioxygenase, benzoate 1,2-dioxygenase, phthalate 4, 5 dioxygenase oxygenase, benzoylformate decarboxylase, alkene monooxygenase), complex polysaccharide degradation (alpha/beta galactosidase, Exo beta 1,3 glucanase, beta hexosaminidase, endo 1,4 beta xylanase.B), beta oxidation (enoyl-CoA hydratase, acyl-CoA dehydrogenase), and Wood–Ljungdahl pathways (carbon monoxide dehydrogenase medium chain; carbon monoxide dehydrogenase large chain; carbon monoxide dehydrogenase small chain) were significantly (*q*-value <0.05) enriched in Fe-amended setups across the different depths (Supplementary Fig. SS7). The enrichment of complex polysaccharide, lignocellulolytic, and aromatic hydrocarbon degrading genes in the Fe-amended setups is in line with the reduction in the FTIR peak for aromatic and carbohydrate moieties compared to the unamended setup (Fig. 3). Thus, the overall functional potential of the community in the Fe amendment setup indicates that both labile and recalcitrant organic carbon fractions were mineralized to CO_2_. These results corroborate gas data in which degradation of these compounds led to increased CO_2_ production (Fig. 2). This is due to the presence of Fe as a TEA that accelerates terminal subdegradation of hydrocarbons and other recalcitrant fractions present in peat to CO_2_ [95].

Furthermore, C1 metabolism varied with depth as well as treatment; formate and methanesulfonate oxidation were more abundant in Fe-amended setups, while methanethiol and methylamine oxidation were highly enriched in unamended setups. However, methane oxidation was highest in lower depth of the unamended setup, suggesting that methane was consumed/oxidized during the incubation over time, as is evident in Fig. 2a.

### Metagenome assembled genomes show distinct metabolic carbon degradation and dissimilatory Fe reduction profiles

To understand the role of the microbes in C/Fe cycling and their associated metabolic capacity, we recovered 120 metagenome-assembled genomes (MAGs) in total with 23 high quality MAGs (>90% completeness and <5% contamination) and 97 moderate quality MAGs (>50% completeness and <10% contamination) (Supplementary Table SS6). Out of these 120 MAGs, 36 belonged to archaeal domain, while 84 were affiliated with bacteria. These MAGs belonged to five archaeal (*Halobacteriota, Micrarchaeota, Nanoarchaeota, Thermoplasmatota, and Thermoproteota*) and 15 bacterial phyla (*Acidobacteriota, Actinomycetota, Bacillota, Bacteroidota, Bdellovirionota, Chlamydiota, Dependentiae, Desulfurobacterota, Elusimicrobiota, Eremiobacterota, FCPU426, Nitrospirota, Patescibacteria, Pseudomonadota, and Verrucomicrobiota)* (Supplementary Table SS6).

**Figure 7:**
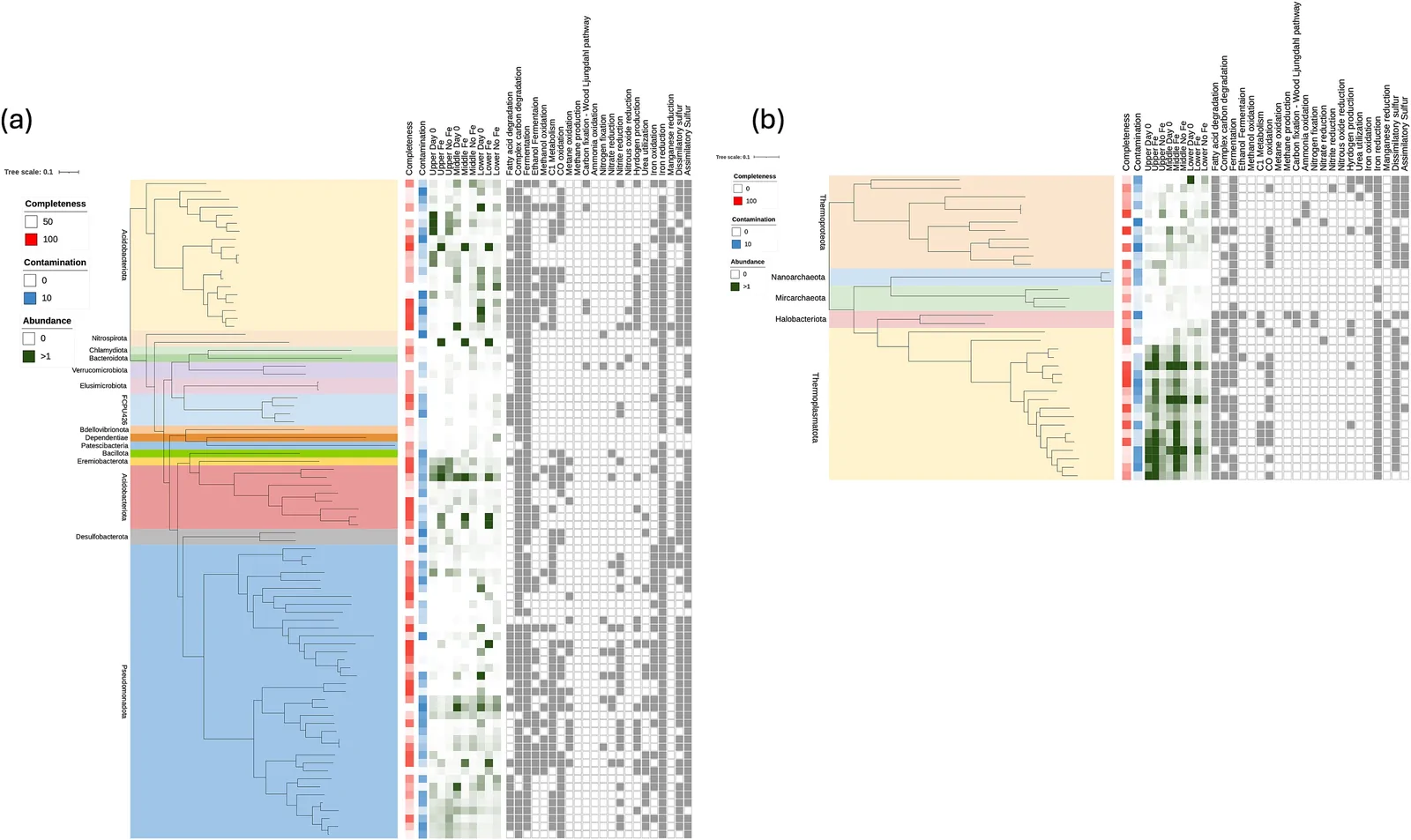
A two-panel figure presenting the phylogenetic diversity and functional characteristics of 120 metagenome-assembled genomes (MAGs) recovered from shotgun metagenomic sequencing of peat samples. Phylogenetic diversity of (a) 84 bacterial and (b) 36 archaeal lineages are represented along with the presence and absence of key metabolic pathways, percentage of completeness, and contamination as well as their relative abundance.

A summary of the key metabolic pathways of the MAGs has been represented in Supplementary Fig. SS7. The abundance pattern of the recovered MAGs showed contrasting trends across the treatments as represented in Fig. 7. Notably, genes involved in C1 metabolism were detected in certain MAGs affiliated to both archaeal and bacterial lineages (Fig. 7a and b). Key genes for methane production (*mcrABC*) and carbon fixation through Wood–Ljungdahl pathway (*cooS, cdhD,* and *cdhE*) were detected in *Methanosarcina* (Bin 45), while the methane monooxygenase (*mmoB*) gene known to assist in the consumption of CH_4_ was found in *Novosphingobium* (Bin 7), *Burkholderiaceae_PKWO01*(Bin 6), and *Gammaproteobacteria_UBA12402* (Bin 1). Interestingly, genes involved in formaldehyde (*fae*) and formate (*fdoG*, *fdhA*) oxidation were detected in *Halobacteriota_bog38* (Bin 75), *Stellaceae_AP15* (Bin 88), *Novosphingobium* (Bin 7 and Bin 10), *Thiomonas* (Bin 3), *Burkholderiacea*-PKWO01(Bin 6), and *Aquitalea* (Bin 9). Genes related to methanol oxidation were detected in *Novosphingobium* (Bin 7 and Bin 10), *Acetobacteraceae_JAJZVS01* (Bin 110), and *Aquitalea* (Bin 9) MAGs. These methylotrophic and methanogenic MAGs have been previously reported from mires and wetlands [96, 97]. The presence of these genes in diverse taxa indicates the phylogenetic extent of the methylotrophy in tropical peatlands, which might be due to the presence of diverse methylated compounds in the peat. Interestingly, increase in abundance of methylotrophic members in *16S rRNA* gene amplicon data (Fig. 5) correlates with the detection of the methylotrophic MAGs belonging to *Methylocystis* (Bin 81, Bin 40 and Bin 107) and *Methylovirgula* (Bin 85) in unamended setups, thereby indicating that Fe(III) amendment does not support methylotrophy. This further corroborates the reduction in CH_4_ production in lower depth unamended setup (Fig. 2). On the other hand, we recovered only two methanogenic MAGs; *Methanosarcina* (a versatile methanogen that can perform both acetoclastic and hydrogenotrophic methanogenesis as well as capable of performing methylotrophic methanogenesis) and Bog-38, also known as *Methanoflorens* (a hydrogenotrophic methanogen) previously reported from permafrost, mires, and wetlands [96–100].

To understand the organic carbon degradation potential of the detected MAGs, genes involved in carbon degradation were identified, as shown in Fig. 7. A summary of these CAZyme gene families (carbohydrate degrading enzymes) belonging to carbohydrate esterases, glycoside hydrolases, and auxiliary activities present in the MAGs has been highlighted in Supplementary Fig. SS8. The presence of auxiliary gene families such as *AA3*, *AA4*, and *AA6* in the members of *Pseudomonadota*, *Actinomycetota*, and *Acidobacteriota* infers their potential toward lignin degradation. Similarly, MAGs harboring glycoside hydrolases families such as GH3, GH13, GH23, and GH103 provide insights about complex polysaccharide degradation from plants and bacteria (Supplementary Fig. SS8) [101, 102].

Additionally, genes known to play a role in Fe reduction, i.e. *OmcF, FmnA, DmkA, FmnB, Ndh2, DmkB, DFE-0448, DFE-0449, DFE-0451, DFE-0461, DFE-0462, DFE-0463, DFE-0464*, and *DFE-0465* were identified in MAGs across different lineages (Supplementary Fig. SS6). The *DmkB* gene was present in a high number of MAGs (110 MAGs) highlighting the importance of Fe reduction as a source of energy for microbes in these tropical peatlands. This suggests that Fe reduction is coupled with organic carbon degradation via fermentation, as validated through CO_2_ production (Fig. 2).

### Limitations of this study

Our study offers valuable insights into the role of Fe (III) as a TEA in C-GHG production, but several limitations persist. Firstly, the microcosm experiment was conducted in a controlled setting, which may not fully mimic natural conditions, possibly impacting ecological interactions. The study did not include a microbial kill control microcosm setup to distinguish the abiotic processes that may occur because of Fe(III) amendment. Secondly, the samples used in the microcosm were from an *Acacia* plantation maintained at −40 cm water table, which is not a reflection of natural peatlands and their hydrological fluctuations. Thirdly, the short 6-week study duration may constrain the scope of the findings. Fourthly, this study cannot be extrapolated to other land uses and estimating actual field-scale emission factors for *Acacia* plantations on tropical peat requires further validation. Nonetheless, these findings need further corroboration through long term field trials to ensure broader ecological relevance of Fe(III) in carbon transformations under natural conditions.

## Conclusion

This controlled microcosm study demonstrates that Fe(III) stimulates C-GHG production in tropical peatlands by promoting organic carbon decomposition. The generation of CO_2_ and CH_4_ varied with depth due to the variation in the initial microbial diversity and the type of organic substrates. This study highlights the underestimated role of Fe(III)-mediated carbon decomposition in peatlands. Therefore, Fe(III) can play a dual role in both stabilizing carbon by binding to it and/or decomposing carbon by functioning as a TEA for iron reducing microbes under oxygen-limiting conditions. Such oxygen-limiting conditions stimulate the redox processes of fermentative and syntrophic bacteria as well. Water table fluctuations associated with the drying and wetting cycle in field conditions will likely stimulate the Fe(III) mediated carbon decomposition and thus subsidence of peatlands. Therefore, reducing the drying–wetting cycle could possibly be considered in the mitigation strategy to manage carbon loss in peatlands.

## Supplementary Material

Supplementary_material_ycag139

## Data Availability

Amplicon and shotgun metagenome raw reads were submitted to NCBI under Bioproject No. PRJNA1040002.

## References

[1] Minasny B, Adetsu DV, Aitkenhead M et al. Mapping and monitoring peatland conditions from global to field scale. *Biogeochemistry* 2024;167:383–425. 10.1007/s10533-023-01084-1

[2] Sasmito SD, Taillardat P, Adinugroho WC et al. Half of land use carbon emissions in Southeast Asia can be mitigated through peat swamp forest and mangrove conservation and restoration. *Nat Commun* 2025;16:740. 10.1038/s41467-025-55892-0 PMC11775092 39875368

[3] Ribeiro K, Pacheco FS, Ferreira JW et al. Tropical peatlands and their contribution to the global carbon cycle and climate change. *Glob Chang Biol* 2021;27:489–505. 10.1111/gcb.15408 33070397

[4] Kiely L, Spracklen DV, Arnold SR et al. Assessing costs of Indonesian fires and the benefits of restoring peatland. *Nat Commun* 2021;12:7044. 10.1038/s41467-021-27353-x PMC8639972 34857766

[5] Noon ML, Goldstein A, Ledezma JC et al. Mapping the irrecoverable carbon in Earth’s ecosystems. *Nat Sustain* 2022;5:37–46. 10.1038/s41893-021-00803-6

[6] Poulter B, Fluet-Chouinard E, Hugelius G et al. A review of global wetland carbon stocks and management challenges. In: Krauss KW, Zhu Z, Stagg CL. (eds), Wetland Carbon and Environmental Management Geophysical Monograph Series, American Geophysical Uion Advancing Earth and Space Sciences (USA) 2021, 1–20. 10.1002/9781119639305.ch1

[7] Beaulne J, Garneau M, Magnan G et al. Peat deposits store more carbon than trees in forested peatlands of the boreal biome. *Sci Rep* 2021;11:2657. 10.1038/s41598-021-82004-x PMC7846601 33514778

[8] Miettinen J, Shi C, Liew SC. Land cover distribution in the peatlands of peninsular Malaysia, Sumatra and Borneo in 2015 with changes since 1990. *Glob Ecol Conserv* 2016;6:67–78. 10.1016/j.gecco.2016.02.004

[9] Page SE, Rieley JO, Banks CJ. Global and regional importance of the tropical peatland carbon pool. *Glob Chang Biol* 2011;17:798–818. 10.1111/j.1365-2486.2010.02279.x

[10] Page S, Mishra S, Agus F et al. Anthropogenic impacts on lowland tropical peatland biogeochemistry. *Nat Rev Earth Environ* 2022;3:426–43. 10.1038/s43017-022-00289-6

[11] Asyhari A, Gangga A, Putra CAS et al. Quantifying the fluxes of carbon loss from an undrained tropical peatland ecosystem in Indonesia. *Sci Rep* 2024;14:11459. 10.1038/s41598-024-62233-6 PMC11106321 38769331

[12] Bowen JC, Hoyt AM, Xu X et al. Aquatic processing enhances the loss of aged carbon from drained and burned peatlands. *Glob Chang Biol* 2024;30:e17394. 10.1111/gcb.17394 38988095

[13] Li Y, Henrion M, Moore A et al. Factors controlling peat soil thickness and carbon storage in temperate peatlands based on UAV high-resolution remote sensing. *Geoderma* 2024;449:117009. 10.1016/j.geoderma.2024.117009

[14] Rydin H, Jeglum JK, Rydin H et al. 77Peat and organic soil. 2006 [cited 11/26/2024]. In: The Biology of Peatlands [Internet]. Oxford University Press, [cited 11/26/2024]; [0]. Available from: 10.1093/acprof:oso/9780198528722.003.0005

[15] Deshmukh CS, Susanto AP, Nardi N et al. Net greenhouse gas balance of fibre wood plantation on peat in Indonesia. *Nature* 2023;616:740–6. 10.1038/s41586-023-05860-9 PMC10132972 37020018

[16] Pickard AE, Heal KV, McLeod AR et al. Temporal changes in photoreactivity of dissolved organic carbon and implications for aquatic carbon fluxes from peatlands. *Biogeosciences* 2017;14:1793–809. 10.5194/bg-14-1793-2017

[17] Dorrepaal E, Toet S, van Logtestijn RSP et al. Carbon respiration from subsurface peat accelerated by climate warming in the subarctic. *Nature* 2009;460:616–9. 10.1038/nature08216

[18] Fenchel T, Blackburn H, King GM. Bacterial Biogeochemistry: The Ecophysiology of Mineral Cycling. Academic press (USA), 2012.

[19] Guth P, Gao C, Knorr K-H. Electron accepting capacities of a wide variety of peat materials from around the globe similarly explain CO2 and CH4 formation. *Glob Biogeochem Cycles* 2023;37:e2022GB007459. 10.1029/2022GB007459

[20] Song T, Liu Y, Kolton M et al. Porewater constituents inhibit microbially mediated greenhouse gas production (GHG) and regulate the response of soil organic matter decomposition to warming in anoxic peat from a sphagnum-dominated bog. *FEMS Microbiol Ecol* 2023;99:fiad060. 10.1093/femsec/fiad060 37280172

[21] AminiTabrizi R, Graf-Grachet N, Chu RK et al. Microbial sensitivity to temperature and sulfate deposition modulates greenhouse gas emissions from peat soils. *Glob Chang Biol* 2023;29:1951–70. 10.1111/gcb.16614 36740729

[22] Patzner MS, Logan M, McKenna AM et al. Microbial iron cycling during palsa hillslope collapse promotes greenhouse gas emissions before complete permafrost thaw. *Commun Earth Environ* 2022;3:76. 10.1038/s43247-022-00407-8

[23] Duchesneau K, Aldeguer-Riquelme B, Petro C et al. Northern peatland microbial communities exhibit resistance to warming and acquire electron acceptors from soil organic matter. *Nat Commun* 2025;16:6869. 10.1038/s41467-025-61664-7 PMC12296737 40715043

[24] Conrad R . Methane production in soil environments—anaerobic biogeochemistry and microbial life between flooding and desiccation. *Microorganisms* 2020;8:881. 10.3390/microorganisms8060881 PMC7357154 32545191

[25] Beal EJ, House CH, Orphan VJ. Manganese- and iron-dependent marine methane oxidation. *Science* 2009;325:184–7. 10.1126/science.1169984 19589998

[26] Valenzuela EI, Prieto-Davó A, López-Lozano NE et al. Anaerobic methane oxidation driven by microbial reduction of natural organic matter in a tropical wetland. *Appl Environ Microbiol* 2017;83:e00645–17. 10.1128/AEM.00645-17 PMC5440706 28341676

[27] Hornibrook ERC, Longstaffe FJ, Fyfe WS. Evolution of stable carbon isotope compositions for methane and carbon dioxide in freshwater wetlands and other anaerobic environments. *Geochim Cosmochim Acta* 2000;64:1013–27. 10.1016/S0016-7037(99)00321-X

[28] Hornibrook ERC . The stable carbon isotope composition of methane produced and emitted from northern peatlands. In: Baird A, Belyea L, Comas X, Reeve A, Slater L. (eds), Carbon Cycling in Northern Peatlands. Geophysical Monograph Series, American Geophysical Union (USA), 2009, 187–203.

[29] Moore TR, Dalva M. Methane and carbon dioxide exchange potentials of peat soils in aerobic and anaerobic laboratory incubations. *Soil Biol Biochem* 1997;29:1157–64. 10.1016/S0038-0717(97)00037-0

[30] Conrad R . Contribution of hydrogen to methane production and control of hydrogen concentrations in methanogenic soils and sediments. *FEMS Microbiol Ecol* 1999;28:193–202. 10.1111/j.1574-6941.1999.tb00575.x

[31] Hausmann B, Knorr K-H, Schreck K et al. Consortia of low-abundance bacteria drive sulfate reduction-dependent degradation of fermentation products in peat soil microcosms. *ISME J* 2016;10:2365–75. 10.1038/ismej.2016.42 PMC4930147 27015005

[32] Raiswell R, Canfield DE. The iron biogeochemical cycle past and present. *Geochem Perspect* 2012;1:1–220. 10.7185/geochempersp.1.1

[33] Yang L, Jiang M, Zou Y et al. Geographical distribution of iron redox cycling bacterial Community in Peatlands: distinct assemble mechanism across environmental gradient. *Front Microbiol* 2021;12:12. 10.3389/fmicb.2021.674411 PMC8185058 34113332

[34] Küsel K, Blöthe M, Schulz D et al. Microbial reduction of iron and porewater biogeochemistry in acidic peatlands. *Biogeosciences* 2008;5:1537–49. 10.5194/bg-5-1537-2008

[35] Patzner MS, Mueller CW, Malusova M et al. Iron mineral dissolution releases iron and associated organic carbon during permafrost thaw. *Nat Commun* 2020;11:6329. 10.1038/s41467-020-20102-6 PMC7729879 33303752

[36] Romanowicz KJ, Crump BC, Kling GW. Genomic evidence that microbial carbon degradation is dominated by iron redox metabolism in thawing permafrost. *ISME Commun* 2023;3:124. 10.1038/s43705-023-00326-5 PMC10667234 37996661

[37] Arun A, Raja PP, Arthi R et al. Polycyclic aromatic hydrocarbons (PAHs) biodegradation by basidiomycetes fungi, pseudomonas isolate, and their cocultures: comparative in vivo and in silico approach. *Appl Biochem Biotechnol* 2008;151:132–42. 10.1007/s12010-008-8160-0 18975143

[38] Bowen JC, Wahyudio PJ, Anshari GZ et al. Canal networks regulate aquatic losses of carbon from degraded tropical peatlands. *Nat Geosci* 2024;17:213–8. 10.1038/s41561-024-01383-8

[39] Huang X, Liu X, Liu J et al. Iron-bound organic carbon and their determinants in peatlands of China. *Geoderma* 2021;391:114974. 10.1016/j.geoderma.2021.114974

[40] Laloo AE, Gupta A, Verrone V et al. Role of Fe and Mn in organo-mineral-microbe interactions: evidence of carbon stabilization and transformation of organic matter leading to carbon greenhouse gas emissions. *Lett Appl Microbiol* 2025;78:ovaf044. 10.1093/lambio/ovaf044 40118507

[41] Huang X, Liu X, Chen L et al. Iron-bound organic carbon dynamics in peatland profiles: the preservation equivalence of deep and surface soil. *Fundam Res* 2023;3:852–60. 10.1016/j.fmre.2022.09.026 PMC11197498 38932997

[42] Maassen S, Balla D, Kalettka T et al. Screening of prevailing processes that drive surface water quality of running waters in a cultivated wetland region of Germany—a multivariate approach. *Sci Total Environ* 2012;438:154–65. 10.1016/j.scitotenv.2012.08.070 23000467

[43] Yang Y, Cheng X, Rene ER et al. Effect of iron sources on methane production and phosphorous transformation in an anaerobic digestion system of waste activated sludge. *Bioresour Technol* 2024;395:130315. 10.1016/j.biortech.2024.130315 38215887

[44] Aromokeye DA, Oni OE, Tebben J et al. Crystalline iron oxides stimulate methanogenic benzoate degradation in marine sediment-derived enrichment cultures. *ISME J* 2021;15:965–80. 10.1038/s41396-020-00824-7 PMC8115662 33154547

[45] Aromokeye DA, Richter-Heitmann T, Oni OE et al. Temperature controls crystalline iron oxide utilization by microbial communities in methanic ferruginous marine sediment incubations. *Front Microbiol* 2018;9:2574. 10.3389/fmicb.2018.02574 PMC6218420 30425692

[46] Zhu X, Yuan Y, Wei X et al. Dissimilatory iron reduction and potential methane production in Chagan Lake wetland soils with carbon addition. *Wetl Ecol Manag* 2021;29:369–79. 10.1007/s11273-021-09783-y

[47] Huang W, Hall SJ. Elevated moisture stimulates carbon loss from mineral soils by releasing protected organic matter. *Nat Commun* 2017;8:1774. 10.1038/s41467-017-01998-z PMC5701196 29176688

[48] Qin L, Freeman C, Zou Y et al. An iron-reduction-mediated cascade mechanism increases the risk of carbon loss from mineral-rich peatlands. *Appl Soil Ecol* 2022;172:104361. 10.1016/j.apsoil.2021.104361

[49] Couwenberg J, Dommain R, Joosten H. Greenhouse gas fluxes from tropical peatlands in South-East Asia. *Glob Chang Biol* 2010;16:1715–32. 10.1111/j.1365-2486.2009.02016.x

[50] Kügler S, Cooper RE, Wegner C-E et al. Iron-organic matter complexes accelerate microbial iron cycling in an iron-rich fen. *Sci Total Environ* 2019;646:972–88. 10.1016/j.scitotenv.2018.07.258 30235650

[51] Bhattacharyya A, Schmidt MP, Stavitski E et al. Iron speciation in peats: chemical and spectroscopic evidence for the co-occurrence of ferric and ferrous iron in organic complexes and mineral precipitates. *Org Geochem* 2018;115:124–37. 10.1016/j.orggeochem.2017.10.012

[52] Kunarso A, Bonner MTL, Blanch EW et al. Differences in tropical peat soil physical and chemical properties under different land uses: a systematic review and meta-analysis. *J Soil Sci Plant Nutr* 2022;22:4063–83. 10.1007/s42729-022-01008-2

[53] Chen C, Hall SJ, Coward E et al. Iron-mediated organic matter decomposition in humid soils can counteract protection. *Nat Commun* 2020;11:2255. 10.1038/s41467-020-16071-5 PMC7206102 32382079

[54] Emsens W-J, Aggenbach CJS, Schoutens K et al. Soil iron content as a predictor of carbon and nutrient mobilization in rewetted fens. *PLoS One* 2016;11:e0153166. 10.1371/journal.pone.0153166 PMC4822970 27050837

[55] Girkin NT, Dhandapani S, Evers S et al. Interactions between labile carbon, temperature and land use regulate carbon dioxide and methane production in tropical peat. *Biogeochemistry* 2020;147:87–97. 10.1007/s10533-019-00632-y

[56] Taillardat P, Moore J, Sasmito S et al. Methane and carbon dioxide production and emission pathways in the belowground and draining water bodies of a tropical peatland plantation forest. *Geophys Res Lett* 2025;52:e2024GL112903. 10.1029/2024GL112903

[57] de Jong AEE, Guererro-Cruz S, van Diggelen JMH et al. Changes in microbial community composition, activity, and greenhouse gas production upon inundation of drained iron-rich peat soils. *Soil Biol Biochem* 2020;149:107862. 10.1016/j.soilbio.2020.107862

[58] Van J Diggelen, L Lamers, J Loermans et al. Towards more Sustainable Hydrological Management and Land Use of Drained Coastal Peatlands-a Biogeochemical Balancing Act, Mires and Peat Vol. 26. 2020. 10.19189/MAP.2019.APG.STA.1771

[59] Ishikura K, Hirata R, Hirano T et al. Carbon dioxide and methane emissions from peat soil in an undrained tropical peat swamp forest. *Ecosystems* 2019;22:1852–68. 10.1007/s10021-019-00376-8

[60] Busman NA, Melling L, Goh KJ et al. Soil CO_2_ and CH_4_ fluxes from different forest types in tropical peat swamp forest. *Sci Total Environ* 2023;858:159973. 10.1016/j.scitotenv.2022.159973 36347298

[61] Prananto JA, Minasny B, Comeau LP et al. Drainage increases CO_2_ and N_2_O emissions from tropical peat soils. *Glob Chang Biol* 2020;26:4583–600. 10.1111/gcb.15147 32391633

[62] Hodgkins SB, Richardson CJ, Dommain R et al. Tropical peatland carbon storage linked to global latitudinal trends in peat recalcitrance. *Nat Commun* 2018;9:3640. 10.1038/s41467-018-06050-2 PMC6128871 30194308

[63] Crow SE, Sulzman EW, Rugh WD et al. Isotopic analysis of respired CO_2_ during decomposition of separated soil organic matter pools. *Soil Biol Biochem* 2006;38:3279–91. 10.1016/j.soilbio.2006.04.007

[64] Chen P, Yuan X-L, Li L-Y et al. Aggregational differentiation of soil-respired CO_2_ and its δ^13^C variation across land-use types. *Geoderma* 2023;432:116384. 10.1016/j.geoderma.2023.116384

[65] Fernandez I, Mahieu N, Cadisch G. Carbon isotopic fractionation during decomposition of plant materials of different quality. *Glob Biogeochem Cycles* 2003;17:1075. 10.1029/2001GB001834

[66] Drollinger S, Knorr K-H, Knierzinger W et al. Peat decomposition proxies of alpine bogs along a degradation gradient. *Geoderma* 2020;369:114331. 10.1016/j.geoderma.2020.114331

[67] Chapman S, Campbell C, Fraser AR et al. FTIR spectroscopy of peat in and bordering Scots pine woodland: relationship with chemical and biological properties. *Soil Biol Biochem* 2001;33:1193–200. 10.1016/S0038-0717(01)00023-2

[68] Cortizas AM, Sjöström JK, Ryberg EE et al. 9000 years of changes in peat organic matter composition in store Mosse (Sweden) traced using FTIR-ATR. *Boreas* 2021;50:1161–78. 10.1111/bor.12527

[69] Joergensen RG . Amino sugars as specific indices for fungal and bacterial residues in soil. *Biol Fertil Soils* 2018;54:559–68. 10.1007/s00374-018-1288-3

[70] Zhang H, Guo Y, Song C et al. Six-year warming decreased amino sugar accumulation in the deep rhizosphere soil of permafrost peatland. *Appl Soil Ecol* 2022;171:104316. 10.1016/j.apsoil.2021.104316

[71] Gadol HJ, Elsherbini J, Kocar BD. Methanogen productivity and microbial community composition varies with iron oxide mineralogy. *Front Microbiol* 2021;12:705501. 10.3389/fmicb.2021.705501 PMC8894893 35250895

[72] Bridge TAM, Johnson DB. Reduction of soluble iron and reductive dissolution of ferric iron-containing minerals by moderately thermophilic iron-oxidizing bacteria. *Appl Environ Microbiol* 1998;64:2181–6. 10.1128/AEM.64.6.2181-2186.1998 PMC106296 9603832

[73] Malik L, Hedrich S. Ferric iron reduction in extreme acidophiles. *Front Microbiol* 2022;12:818414. 10.3389/fmicb.2021.818414 PMC8790237 35095822

[74] Gupta A, Dutta A, Sarkar J et al. Low-abundance members of the *Firmicutes* facilitate bioremediation of soil impacted by highly acidic mine drainage from the Malanjkhand copper project, India. *Front Microbiol* 2018;9:2882. 10.3389/fmicb.2018.02882 PMC6297179 30619102

[75] Christiansen NA, Green TJ, Fryirs KA et al. Bacterial communities in peat swamps reflect changes associated with catchment urbanisation. *Urban Ecosyst* 2022;25:1455–68. 10.1007/s11252-022-01238-3

[76] Azizan SNF, Murakami S, McTaggart I et al. Changes in specific microbial groups characterize the impact of land conversion to oil palm plantations on peat. *Front For Glob Change* 2024;7:1305491. 10.3389/ffgc.2024

[77] Kirschke S, Bousquet P, Ciais P et al. Three decades of global methane sources and sinks. *Nat Geosci* 2013;6:813–23. 10.1038/ngeo1955

[78] Wang H, Weil M, Zak D et al. Temporal and spatial dynamics of peat microbiomes in drained and rewetted soils of three temperate peatlands. bioRxiv. 2020:2020.02.16.951285.

[79] Robroek BJM, Martí M, Svensson BH et al. Rewiring of peatland plant–microbe networks outpaces species turnover. *Oikos* 2021;130:339–53. 10.1111/oik.07635

[80] Peng Q-a, Shaaban M, Wu Y et al. The diversity of iron reducing bacteria communities in subtropical paddy soils of China. *Appl Soil Ecol* 2016;101:20–7. 10.1016/j.apsoil.2016.01.012

[81] Mayilraj S, Ruckmani A, Kaur C et al. A novel member of the genus *Cohnella* isolated from haematite ore. *Curr Microbiol* 2011;62:1704–9. 10.1007/s00284-011-9917-1 21409616

[82] Xue W, Ma H, Deng K et al. Substrates-mediated microbes mitigate carbon loss in shrub peatlands. *J Plant Ecol* 2025;18:1.

[83] Kulichevskaya IS, Danilova OV, Tereshina VM et al. Descriptions of *Roseiarcus fermentans* gen. nov., sp. nov., a bacteriochlorophyll a—containing fermentative bacterium related phylogenetically to alphaproteobacterial methanotrophs, and of the family *Roseiarcaceae* fam. nov. *Int J Syst Evol Microbiol* 2014;64:2558–65. 10.1099/ijs.0.064576-0 24812364

[84] Gu Y, Bai Y, Xiang Q et al. Degradation shaped bacterial and archaeal communities with predictable taxa and their association patterns in Zoige wetland at Tibet plateau. *Sci Rep* 2018;8:3884. 10.1038/s41598-018-21874-0 PMC5832768 29497087

[85] Schmidt O, Horn MA, Kolb S et al. Temperature impacts differentially on the methanogenic food web of cellulose-supplemented peatland soil. *Environ Microbiol* 2015;17:720–34. 10.1111/1462-2920.12507 24813682

[86] Xiang X, Wang H, Man B et al. Diverse *Bathyarchaeotal* lineages dominate archaeal communities in the acidic Dajiuhu peatland. *Central China Microb Ecol* 2023;85:557–71. 10.1007/s00248-022-01990-1 35332366

[87] Bräuer SL, Basiliko N, Siljanen HMP et al. Methanogenic archaea in peatlands. *FEMS Microbiol Lett* 2020;367:fnaa172. 10.1093/femsle/fnaa172 33068423

[88] Hu L, Long Y, Fang C. Effect of dissimilatory iron reduction on the reduction of CH4 production in landfill conditions. *J Chem* 2019;2019:5163132. 10.1155/2019/5163132

[89] Zhou S, Xu J, Yang G et al. Methanogenesis affected by the co-occurrence of iron(III) oxides and humic substances. *FEMS Microbiol Ecol* 2014;88:107–20. 10.1111/1574-6941.12274 24372096

[90] Samanides CG, Koutsokeras L, Constantinides G et al. Methanogenesis inhibition in anaerobic granular sludge for the generation of volatile fatty acids from CO_2_ and zero valent iron. *Front Energy Res* 2020;8:37. 10.3389/fenrg.2020.00037

[91] Teh YA, Dubinsky EA, Silver WL et al. Suppression of methanogenesis by dissimilatory Fe(III)-reducing bacteria in tropical rain forest soils: implications for ecosystem methane flux. *Glob Chang Biol* 2008;14:413–22. 10.1111/j.1365-2486.2007.01487.x

[92] Zhan W, Tian Y, Zhang J et al. Mechanistic insights into the roles of ferric chloride on methane production in anaerobic digestion of waste activated sludge. *J Clean Prod* 2021;296:126527. 10.1016/j.jclepro.2021.126527

[93] Wilson RM, Tfaily MM, Kolton M et al. Soil metabolome response to whole-ecosystem warming at the spruce and peatland responses under changing environments experiment. *Proc Natl Acad Sci U S A* 2021;118:e2004192118. 10.1073/pnas.2004192118 PMC8237682 34161254

[94] Yule CM, Lim YY, Lim TY. Recycling of phenolic compounds in Borneo’s tropical peat swamp forests. *Carbon Balance Manag* 2018;13:3. 10.1186/s13021-018-0092-6 PMC5803172 29417248

[95] Zhang X, Xue W, Wang G et al. Biogeochemical coupling of C/Fe in oil-polluted wetlands associated with iron reduction. *Commun Earth Environ* 2025;6:77. 10.1038/s43247-025-02062-1

[96] Ellenbogen JB, Borton MA, McGivern BB et al. Methylotrophy in the mire: direct and indirect routes for methane production in thawing permafrost. *mSystems* 2023;9:e00698–23. 10.1128/msystems.00698-23 PMC10805028 38063415

[97] Reji L, Duan J, Myneni SCB et al. Distinct microbiomes underlie divergent responses of methane emissions from diverse wetland soils to oxygen shifts. *ISME Commun* 2025;5:ycaf063. 10.1093/ismeco/ycaf063 PMC12075768 40371176

[98] Mondav R, Woodcroft BJ, Kim E-H et al. Discovery of a novel methanogen prevalent in thawing permafrost. *Nat Commun* 2014;5:3212. 10.1038/ncomms4212 24526077

[99] McCalley CK, Woodcroft BJ, Hodgkins SB et al. Methane dynamics regulated by microbial community response to permafrost thaw. *Nature* 2014;514:478–81. 10.1038/nature13798 25341787

[100] Woodcroft BJ, Singleton CM, Boyd JA et al. Genome-centric view of carbon processing in thawing permafrost. *Nature* 2018;560:49–54. 10.1038/s41586-018-0338-1 30013118

[101] Hausmann B, Pelikan C, Herbold CW et al. Peatland *Acidobacteria* with a dissimilatory sulfur metabolism. *ISME J* 2018;12:1729–42. 10.1038/s41396-018-0077-1 PMC6018796 29476143

[102] Xiong M, Jiang W, Zou S et al. Microbial carbohydrate-active enzymes influence soil carbon by regulating the of plant- and fungal-derived biomass decomposition in plateau peat wetlands under differing water conditions. *Front Microbiol* 2023;14: 1266016. 10.3389/fmicb.2023.1266016 PMC10507343 37731925

