# Supplementary material for: Iron stimulates greenhouse gas production in Southeast Asian peatlands
Source: ISME Commun. 2026 Jun 5;6(1):ycag139. doi: 10.1093/ismeco/ycag139 (PMC13600443; doi:10.1093/ismeco/ycag139)
Supplement: Supplementary file 1 [file supplementary_material_ycag139.zip › Supplementary_File_30-4-2026.docx]

Supplementary Information

**Iron stimulates greenhouse gas production in Southeast Asian peatlands**

Andrew Elohim Laloo^a,b#^, Abhishek Gupta^a,b#^, Valeria Verrone^a^, Rama Kant Dubey^a,c^, Pierre Taillardat^a,d^, Rajpirathap Nagasaijanani^b^, Muhammad Iman Faisal Harahap^e^, Agnieszka M Banas^g^, Krzysztof Banas^g^, Raktim Bhattacharya^a^, Sophie Lok Sze Min^a^, Yi Zi Koh^b^, Fairuz Razali^a^, Nikken Irfa Nastiti^f^, Eka Sri Kandi Putri^f^, Sofyan Kurnianto^e^, Chandra Shekhar Deshmukh^e^, Romy Chakraborty^h^, Sanjay Swarup*^a,b,c^.

^a^National University of Singapore Environmental Research Institute, National University of Singapore,117411, Singapore

^b^Singapore Centre for Environmental Life Sciences Engineering, National University of Singapore, Singapore

^c^Department of Biological Sciences, National University of Singapore, 117558, Singapore

^d^ Asian School of the Environment, Nanyang Technological University, 639798, Singapore.

^e^ Asia Pacific Resources International Ltd. Pelalawan Regency, Indonesia

^f^ Department of Silviculture, IPB University, Bogor, 16680, Indonesia.

^g^ Singapore Synchrotron Light Source, National University of Singapore, 5 Research Link, Singapore 117603, Singapore.

^h^ Department of Ecology, Earth and Environmental Sciences Area, Lawrence Berkeley National Laboratory, Berkeley, CA 94720, United States of America.

* Corresponding author -  [sanjay@nus.edu.sg](mailto:sanjay@nus.edu.sg)

^#^ Equal contributing author

**Supplementary Material and Method.**

**Geochemical analysis**

Slurry samples were passed through a 0.22 μM syringe filter and the obtained filtrate was used for estimation of Fe(II) anions, and heavy metals. Samples for heavy metal estimation were acidified with HNO_3_, while 0.1N HCl was added in the sample’s aliquot for Fe(II) estimation. Additionally, Fe(II) estimation was performed using the standardized Ferrozine method (1). However, high levels of humic substances hindered the collection of reliable colorimetric data, hence the results are not provided in this study. Metals and anions were analyzed by Inductively Coupled Mass Spectrometry (ICP-MS) and Ion Chromatography (IC), respectively.

**Greenhouse gases CH_4_ and CO_2_ estimation:**

Gas samples were collected from the headspace of the serum vials by piercing the rubber stop with a syringe and injecting the gas in pre-evacuated airtight 13mL Exetainer vials (Labco, UK). Gas samples for CO_2_ and CH_4_ concentrations and stable isotope values (δ^13^C-CO_2_ and δ^13^C-CH_4_) determination were analyzed in the laboratory using a cavity ring-down spectroscopy (CRDS) analyzer (G2201-i, Picarro Inc., USA) equipped with a gas autosampler (SAM, OpenAutosampler Inc., Canada). The isotopic CO_2_ and CH_4_ data are reported in standard data notation (δ) expressed in ‰ relative to the Vienna Pee Dee Belemnite standard. To ensure consistency of the measurements, gas standards (AlphagazTM Isotope Natural Air, Airgas, USA) were analyzed at the beginning, in the middle (after 25 samples), and at the end of each run.

**FTIR analysis**

Samples were analysed by means of Attenuated Total Reflectance Fourier Transform Infrared (ATR-FTIR) spectroscopy at the ISMI beamline facility of the Singapore Synchrotron Light Source (SSLS). Spectra were acquired using a Bruker Vertex 80v vacuum spectrometer equipped with a Globar source, a KBr beamsplitter, and a deuterated L-alanine doped triglycine sulfate (DLaTGS) detector. Measurements were conducted using a mIRage ATR accessory (PIKE Technologies) featuring a ZnSe/diamond crystal (1.6 mm diameter). For each analysis, a 2 µL aliquot of the sample was deposited onto the crystal surface. Data were collected over the 4000–520 cm⁻¹ spectral range under the control of a custom macro in OPUS software (Bruker). A total of 70 consecutive spectra were recorded for each sample, with each individual spectrum resulting from the co-addition of 16 scans acquired over 15 seconds, followed by a 1-second wait time. To prevent carry-over contamination, the ATR crystal was rigorously cleaned between measurements. A fresh background spectrum was recorded from the clean crystal prior to the analysis of each new sample. To ensure data reproducibility and assess sample homogeneity, all samples were analyzed in triplicate.

**Metabolites profiling**

The filtered slurry samples were freeze dried and resuspended in methanol for analysis. Analysis was carried out on day 0 and day 42 (Fe-amended) only. Metabolite analysis was conducted using an Agilent 1290 Ultra High-Performance Liquid Chromatography (UHPLC) system, controlled via Mass Hunter Data Acquisition Online Workstation Software. Metabolites were separated on an Agilent Zorbax RRHD XDB-C18 analytical column (100 mm × 2.1 mm, 1.8 µm) with a corresponding guard column. Chromatographic separation occurred at 40°C with a flow rate of 0.3 mL/min and an injection volume of 5 µL. The mobile phase consisted of a binary gradient system: 0.1% formic acid in water (eluent A) and 0.1% formic acid in LC-MS grade acetonitrile (eluent B). The gradient elution profile was as follows: from 0 to 7 minutes, 98% A and 2% B; from 7 to 17 minutes, 70% A and 30% B; from 17 to 19.1 minutes, 2% A and 98% B; and from 19.1 to 23 minutes, 98% A and 2% B.

Mass spectrometric analysis was performed using an Agilent 6540 Quadrupole Time-of-Flight (QTOF) mass spectrometer, equipped with a dual Agilent Jet Stream (AJS) electrospray ionization (ESI) source, operating in both positive and negative ionization modes. For positive ESI, the source temperature was set to 250°C, while for negative ESI, it was set to 280°C. The gas flow rate was maintained at 10 L/min, with a nebulizer pressure of 35 psi. The sheath gas flow was set at 11 L/min, with a sheath gas temperature of 250°C. The capillary voltage was adjusted to 3500V for positive ESI and 3000 V for negative ESI. Mass spectra were acquired over a scan range of 50–1200 m/z.

The raw mass spectrometric data were analyzed using a previously described method (2). Agilent raw files (in ‘.d’ format) were processed in Agilent MassHunter software (Agilent Technologies, USA) for peak identification, utilizing molecular feature extraction with retention shifts. Identified peaks were matched with the Agilent METLIN Personal Compound Database and Library (PCDL), and compounds were listed against their target peaks as peak volume measurements. The results were exported from the Agilent MassHunter software for further analysis.

**DNA extraction, amplification, and sequencing**

Slurry samples recovered from each of the microcosm setup were subjected to high centrifugation to pellet down the peat. 300 mg of the pelleted peat was used for DNA extraction using FastDNA™ SPIN Kit for Soil (MP Biomedicals) as per the manufacturer’s protocol. The extracted DNA was passed through Zymo-Spin III-HRC Filters (Zymo Research) for 2-3 times to remove the humic acid or polyphenols extracted along with the DNA. The purified DNA was subjected to both shotgun metagenome and 16S rRNA based targeted amplicon sequencing. The V4-V5 region of the 16S rRNA gene was amplified using 515F and 926R primer set (3). The PCR reaction was performed in 30 μl volume which contained 10 ng of DNA template, 0.5 μl BSA (stock 10 mg/ml), 3 μl of primers (10 picomole), 3 μl of dNTPs (2mM), 1X DreamTaq buffer (10X, Thermofisher Scientific), 0.6μl of Dream Taq polymerase (5unit/μl, Thermofisher Scientific). The PCR condition used for its amplification was as follows: initial denaturation at 95^o^C for 5 min followed by 28 cycles of denaturation at 95^o^C for 30 sec, annealing at 50^o^C for 30 sec and extension at 95^o^C for 45 sec with final extension at 72^o^C for 5 min. Two types of negative controls (DNA kit control and PCR negative control) were considered for amplification. The amplified products were run on the 1% agarose gel for 30 min and the PCR products were purified using GeneJET PCR Purification Kit (ThermoFisher Scientifc). The amplicons were sequenced in Illumina MiSeq platform using 300X2 bp chemistry, respectively at Singapore Centre for Environmental Life Sciences Engineering (SCELSE) sequencing facility, Singapore. Shotgun-metagenome sequencing was performed for 27 samples (including day 0, Fe-amended and unamended samples from day 42) in HiSeqX Illumina platform (150X2 bp chemistry).

**16S rRNA gene amplicon sequence analysis**

Raw amplicon sequences obtained after Illumina MiSeq sequencing were subjected to quality check using FastQC (1) followed by pre-processing and analysis using Divisive Amplicon Denoising Algorithm 2 (DADA2) package v1.23.0 (4). Briefly, primers and low-quality bases were removed from the end of the reads using filter and Trim function of DADA2. Non-chimeric, and error free amplicon sequence variants (ASVs) were generated and subjected to taxonomic assignments using SILVA Database (silva_nr-99_v138.1_train_set.fa.gz). Negative control samples were also sequenced and Decontam v1.18.0 package was used to remove contaminants from the main datasets using prevalence-based method (5). Both alpha and beta diversity metrics were generated by Phyloseq v3.4.2 R package (6). To understand the changes in bacterial and archaeal communities separately, reads assigned to bacterial and archaeal domains were segregated and separate analysis was performed for both the domains. Various R packages were used for data visualization such as ggplot2 v3.5.2 (7), vegan v2.6-6.1 (8), and RColorBrewer v1.1-3 (9). Changes in alpha diversity metrics and relative abundance of phyla across the depths were visualized with geom_smooth lm function, while pearson correlation tests were performed using *stat_cor* function within ggpubr v0.6.0 package (10). Principal coordinate analysis (PCoA) was performed with Bray-Curtis's dissimilarity matrix to understand the difference in the community composition between the treatments and experimental setups. Permutational ANOVA (PERMANOVA) with adjusted p-value was performed using Bray-Curtis's dissimilarity matrix to assess the changes in community composition across timepoint, treatment and depth using adonis2 function. Correlations between depth and geochemical parameters were calculated using the Corrplot v0.95 package (11). Correlation values with p<0.05 were considered as significant. Analysis of variance was performed to analyze the difference in the gas production over time in Fe-amended and unamended setups. A heat-map based relative distribution of major bacterial genera in peat samples across three depths during microcosm incubation was generated using pheatmap 1.0.12 package (12). Changes in the relative abundance of microbial genera were tested for temporal differences across different timepoints (Day_0, Day_14, Day_28, Day_42) within each depth and treatment combination using Kruskal–Wallis rank-sum tests. Significant genera (p<0.05) underwent pairwise post-hoc comparisons using Dunn’s test (FDR-adjusted). The relative abundance of the key 23 genera (presented in Figure 5) was visualized over time using ggplot2, with treatment-specific linear trend lines added via geom_smooth(method = "lm") and Spearman correlation coefficients (R) along with their p-values displayed using stat_cor from the ggpubr package (Supplementary Fig.6).

**Metagenome analysis**

The raw reads obtained from 27 samples were processed using Cutadapt v4.1 (13) to remove sequencing adapters followed by trimming of low-quality regions using BBduk (14). The resulted quality control reads from each sample were assembled using a modified workflow described previously (15, 16). Briefly, both MetaSpades v3.13.0 and Megahit v1.2.9 were used for assembly (17, 18). The quality of the assembly was assessed through QUAST v5.2.0 (19). The binning of individual assembly obtained from MetaSpades and Megahit was performed using MetaBAT2 v2.12.1 and MaxBin2 v2.2.7 (20, 21) following which metagenome assembled genomes (MAGs) completeness and contamination were estimated using CheckM2 v1.2.1 (22). Only MAGs which were at least 50% complete and less than 10% contamination were considered for downstream analyses. The MAGs were dereplicated using DAS Tool v1.1.5 (23) and dRep v3.0.0 (24). Taxonomic classification of the final MAGs were performed using GTDB-Tk v2.4.0 (25). Additionally, METABOLIC v4.0 was run on the MAGs to identify key genes that are involved in Carbon and other biogeochemical cycling (26). Abundance or coverage of MAGs were calculated using coverM by mapping the MAGs on the metagenomic reads (27). A phylogenetic tree of the bacterial and archaeal MAGs was constructed using de novo workflow in GTDB-Tk v2.4.0 (28) and visualized and annotated with MAGs completeness, contamination, detection in treatments, and presence of genes to different metabolism using the interactive tree of life software (iTOL v7) (29). Furthermore, to understand the metabolic potential of the entire microbial community, we performed functional annotation using Prokka v1.14.6 on the assembled contigs generated by Megahit assembly (30). Normalization of the protein hit counts was done through counts per million (cpm). Genes related to carbon degradation and other metabolisms were retrieved and presented in the heatmap using pheatmap v1.0.12 package (12). Differentially enriched genes involved in sulfur metabolism, nitrogen metabolism, C1, metabolism, carbon degradation, stress response, hydrogenases, fermentation, and Wood-ljungdahl pathways were identified using MaAsLin2 analysis (analysis method = 'LM', correction='BH')(31). The differentially enriched genes with qvalue less than 0.05 were identified and corresponding -log10(qvalue) were represented in volcano plot

**Carbon degradation potential detected in MAGs.**

One or multiple cellulose and hemicellulose degrading genes were found in MAGs related to *Palsa-1180_JAJPJW01* (Bin 19 and Bin 38), *Novosphingobium* (Bin 7 and Bin 10), *Burkholderiaceae*_PKWO01 (Bin 6), *Aquitalea* (Bin 9), *Leptospirillaceae* (bin 2), *Acidocella* (Bin 25), *Binataceae* (Bin 42 and 94), *Bdellovibrionales* (bin 101), *Thermoplasmatales_GCA-001856825* (Bin 33). Chitin degrading genes were found in several MAGS including *Chlamydiales_CAIPRZ01* (Bin 41), *Elusimicrobia_UBA9628* (Bin 72 and 73), *Tyrphobacter* (Bin 18), *Palsa-1180_JAJPJW01* (Bin 19 and 38), *Leptospirillaceae* (Bin 2), *Leptospirillaceae_JAJYGC01* (Bin 80), *Novosphingobium* (Bin 7 and 10), *Berkiellaceae* (Bin 49), *Thiomonas* (Bin 3), *Burkholdericeae_PKWO01*(Bin 6), *Aquitalea* (Bin 9), *Moraxellaceae* (Bin 29), *Gammaproteobacteria_UBA12402* (Bin 1). Phenol degradation genes were detected in *Halobacteriota_bog38* (Bin 75), *Methanosarcina* (Bin 45), *Thermoplasmatales_GCA-001856825* (Bin 33), *Thermoproteota_FEN987* (Bin 51), *Nitrososphaerales_CADDZS01* (Bin 11), *Nitrosotalea* (Bin 22 and 60), *Tyrphobacter* (Bin 18), *Palsa-1180_JAJPJW01* (Bin 19 and 38), *Stellaceae* (Bin 64), *Stellaceae_AP-15* (Bin 55, 93 and 35 and 78), *Thiomonas* (Bin 3), *Burkholdericaea_PKWO01*(Bin 6), *Aquitalea* (Bin 9), *Pedosphaerales_UBA11358* (Bin 74). These microbial bins indicate their diverse metabolic potential to degrade complex and recalcitrant organic compounds of peat under Fe amended and unamended condition. Genes encoding for acetate production via fermentation i.e. *acdA, ack* and *pta* were detected in most of the MAGs (88 MAGs) across different archaeal and bacterial phyla indicating their fermentative potential as highlighted in Supplementary Fig.6.

**Supplementary Table 1. Change in pH across the treatments over time.**

| **Treatment** | **Depth** | **Day 0** | **Day 14** | **Day 28** | **Day 42** |
| --- | --- | --- | --- | --- | --- |
| No Fe | Upper | 2.70±0.17 | 2.81±0.14 | 2.90±0.07 | 2.96±0.06 |
|  | Middle | 2.71±0.10 | 2.79±0.07 | 2.81±0.10 | 2.87±0.04 |
|  | Lower | 2.76±0.02 | 2.83±0.14 | 2.98±0.14 | 2.95±0.02 |
| Fe | Upper | 2.21±0.14 | 2.24±0.19 | 2.26±0.28 | 2.28±0.06 |
|  | Middle | 2.27±0.07 | 2.25±0.11 | 2.20±0.02 | 2.17±0.10 |
|  | Lower | 2.24±0.23 | 2.29±0.15 | 2.19±0.23 | 2.25±0.05 |

**Supplementary Table 2. Analysis of Variance Table: CH_4_ production**

|  | **Df** | **Sum Sq** | **Mean Sq** | **F value** | **Pr(>F)** | **Significance** |
| --- | --- | --- | --- | --- | --- | --- |
| Timepoints | 3 | 459 | 153 | 0.0954 | 0.96215 |  |
| Treatment | 1 | 55649 | 55649 | 34.7305 | 6.137e-07 | *** |
| Depth | 2 | 161972 | 80986 | 50.5432 | 8.605e-12 | *** |
| Timepoints:Treatment:Depth | 14 | 49444 | 3532 | 2.2041 | 0.02499 | * |
| Residuals | 41 | 65695 | 1602 |  |  |  |
| --- |  |  |  |  |  |  |
| Signif. codes: | 0 ‘***’ | 0.001 ‘**’ | 0.01 ‘*’ | 0.05 ‘.’ | 0.1 ‘ ’ |  |

**Supplementary Table 3. Analysis of Variance Table: CO_2_ production**

|  | **Df** | **Sum Sq** | **Mean Sq** | **F value** | **Pr(>F)** | **Significance** |
| --- | --- | --- | --- | --- | --- | --- |
| Timepoints | 3 | 2.23E+10 | 7435722254 | 38.9397 | 3.37E-12 | *** |
| Treatment | 1 | 8.82E+09 | 8815874176 | 46.1673 | 2.86E-08 | *** |
| Depth | 2 | 1.0367e+09 | 518343004 | 2.7145 | 0.07786 | . |
| Timepoints:Treatment:Depth | 14 | 5.7482e+09 | 410586660 | 2.1502 | 0.02822 | * |
| Residuals | 42 | 8.0201e+09 | 190954939 |  |  |  |
| --- |  |  |  |  |  |  |
| Signif. codes: | 0 ‘***’ | 0.001 ‘**’ | 0.01 ‘*’ | 0.05 ‘.’ | 0.1 ‘ ’ |  |

**Supplementary Table 4: Isotopic Signatures of CH_4_ and CO_2_**

|  |  | ***δ*^13^C -CH_4_ (‰)** | | | ***δ*^13^C -CO_2_ (‰)** | | |
| --- | --- | --- | --- | --- | --- | --- | --- |
| **Layer** | **Timepoint** | **Original Sample** | **Fe-amended** | **Unamended** | **Original Sample** | **Fe-amended** | **Unamended** |
| **Upper** | **Day_0** | -35.786± 3.67 |  |  | -22.007±0.46 |  |  |
|  | **Day_14** |  | ND | ND |  | -26.255± 0.93 | -28.229±1.42 |
|  | **Day_28** |  | ND | ND |  | -23.386±0.01 | -28.290±1.67 |
|  | **Day_42** |  | ND | ND |  | -27.578±0.01 | -28.601±0.34 |
| **Middle** | **Day_0** | -27.293±7.51 |  |  | -22.724±1.81 |  |  |
|  | **Day_14** |  | -27.512±5.66 | ND |  | -27.752±1.89 | -28.781±1.05 |
|  | **Day_28** |  | -21.384±8.89 | ND |  | -28.690±1±11 | -29.762±1.21 |
|  | **Day_42** |  | NA | ND |  | -28.552±1.66 | -29.457±0.33 |
| **Lower** | **Day_0** | -67.656±6.92 |  |  | -22.866±1.40 |  |  |
|  | **Day_14** |  | -69.726±1.76 | -55.731±4.61 |  | -25.661±0.15 | -29.385±1.36 |
|  | **Day_28** |  | -67.787±4.75 | -48.176±1.26 |  | -27.278±0.27 | -30.452±1.28 |
|  | **Day_42** |  | -63.750±4.81 | -37.685±3.95 |  | -28.251±0.12 | -30.077±0.16 |
| Data is presented as average of triplicates with standard deviation  ND: Not detected  NA: Data not available | | | | | | | |

**Supplementary Table 5**: **Fold change in relative abundance of major bacterial phyla at the end of the microcosm incubation study.**

| **Layer** | **Upper** | | **Middle** | | **Lower** | |
| --- | --- | --- | --- | --- | --- | --- |
| **Amendments** | Fe | NoFe | Fe | NoFe | Fe | NoFe |
| *Nitrospirota* | 1470.73965 | 15.5889923 | 14.6659482 | -0.1816477 | 17.4724477 | -0.5019511 |
| *Proteobacteria* | -0.2243931 | -0.1228683 | -0.6198204 | -0.2550074 | -0.4928988 | -0.2637328 |
| *Acidobacteriota* | -0.739957 | -0.3271144 | -0.8853956 | -0.1768057 | -0.6958636 | -0.1292235 |
| *Actinobacteriota* | 4.58116992 | -0.3086656 | 6.07294871 | 0.06006685 | 24.1476916 | 3.19904759 |
| *WPS-2* | 0.65689064 | 1.05568941 | -0.2507858 | 1.29438999 | 2.29720655 | 2.35649676 |
| *Firmicutes* | 28.2477288 | 1.43476542 | 1030.31703 | 13.6372117 | 55.5662891 | 3.13497484 |
| *RCP2-54* | -0.0820691 | 1.56244306 | -0.7930466 | 0.48261756 | -0.5987065 | 0.60702229 |
| *Planctomycetota* | -0.7722063 | 0.61467281 | -0.8250177 | 1.09654706 | -0.4494224 | 0.86765849 |
| *Verrucomicrobiota* | -0.8161447 | 4.39499084 | -0.935305 | 1.9544197 | -0.2857941 | 1.84152324 |
| *Bacteroidota* | -0.9933479 | 9.17875589 | -0.7749256 | 19.2620134 | 3.59267216 | 34.861834 |

**Supplementary Table 6: Metagenome assembled genomes (MAGs) assembled from short gun metagenomics with <50% completeness and >10 contamination.**

| **Bin name** | **Taxonomy** | **Completeness** | **Contamination** |
| --- | --- | --- | --- |
| Bin 75 | d__Archaea;p__Halobacteriota;c__Bog-38;o__Bog-38;f__Bog-38;g__;s__ | 67.84 | 1.71 |
| Bin 45 | d__Archaea;p__Halobacteriota;c__Methanosarcinia;o__Methanosarcinales;f__Methanosarcinaceae;g__Methanosarcina;s__ | 82.04 | 8.93 |
| Bin 59 | d__Archaea;p__Micrarchaeota;c__Micrarchaeia;o__Micrarchaeales;f__Micrarchaeaceae;g__JAJZYD01;s__ | 74.76 | 1.20 |
| Bin 89 | d__Archaea;p__Micrarchaeota;c__Micrarchaeia;o__Micrarchaeales;f__Micrarchaeaceae;g__JAJZYD01;s__ | 59.53 | 0.91 |
| Bin 99 | d__Archaea;p__Micrarchaeota;c__Micrarchaeia;o__Micrarchaeales;f__Micrarchaeaceae;g__JAJZYD01;s__ | 55.79 | 1.30 |
| Bin 83 | d__Archaea;p__Nanoarchaeota;c__Nanoarchaeia;o__Parvarchaeales;f__Parvarchaeaceae;g__JAJYZA01;s__ | 63.71 | 6.31 |
| Bin 56 | d__Archaea;p__Nanoarchaeota;c__Nanoarchaeia;o__Parvarchaeales;f__Parvarchaeaceae;g__JAJYZA01;s__JAJYZA01sp030044235 | 76.56 | 5.61 |
| Bin 33 | d__Archaea;p__Thermoplasmatota;c__Thermoplasmata;o__Thermoplasmatales;f__GCA-001856825;g__GCA-001856825;s__ | 90.59 | 0.88 |
| Bin 30 | d__Archaea;p__Thermoplasmatota;c__Thermoplasmata;o__UBA184;f__UBA184;g__JAJYEA01;s__ | 91.06 | 3.60 |
| Bin 26 | d__Archaea;p__Thermoplasmatota;c__Thermoplasmata;o__UBA184;f__UBA184;g__JAKIWV01;s__ | 92.80 | 3.20 |
| Bin 96 | d__Archaea;p__Thermoplasmatota;c__Thermoplasmata;o__UBA184;f__UBA184;g__JAKIWV01;s__JAKIWV01sp030037265 | 57.45 | 0.00 |
| Bin 109 | d__Archaea;p__Thermoplasmatota;c__Thermoplasmata;o__UBA184;f__UBA184;g__JAKIWV01;s__JAKIWV01sp030037265 | 53.19 | 3.20 |
| Bin 13 | d__Archaea;p__Thermoplasmatota;c__Thermoplasmata;o__UBA184;f__UBA184;g__JAKIWV01;s__JAKIWV01sp030037955 | 97.05 | 8.84 |
| Bin 21 | d__Archaea;p__Thermoplasmatota;c__Thermoplasmata;o__UBA184;f__UBA184;g__JAKIWV01;s__JAKIWV01sp030041175 | 95.50 | 6.40 |
| Bin 27 | d__Archaea;p__Thermoplasmatota;c__Thermoplasmata;o__UBA184;f__UBA184;g__UBA184;s__ | 92.27 | 7.05 |
| Bin 34 | d__Archaea;p__Thermoplasmatota;c__Thermoplasmata;o__UBA184;f__UBA184;g__UBA184;s__ | 90.29 | 2.40 |
| Bin 63 | d__Archaea;p__Thermoplasmatota;c__Thermoplasmata;o__UBA184;f__UBA184;g__UBA184;s__ | 71.97 | 8.69 |
| Bin 84 | d__Archaea;p__Thermoplasmatota;c__Thermoplasmata;o__UBA184;f__UBA184;g__UBA184;s__ | 63.04 | 6.40 |
| Bin 87 | d__Archaea;p__Thermoplasmatota;c__Thermoplasmata;o__UBA184;f__UBA184;g__UBA184;s__ | 61.54 | 2.40 |
| Bin 92 | d__Archaea;p__Thermoplasmatota;c__Thermoplasmata;o__UBA184;f__UBA184;g__UBA184;s__ | 58.64 | 7.79 |
| Bin 71 | d__Archaea;p__Thermoplasmatota;c__Thermoplasmata;o__UBA184;f__UBA184;g__UBA184;s__UBA184sp030037395 | 69.82 | 7.85 |
| Bin 79 | d__Archaea;p__Thermoplasmatota;c__Thermoplasmata;o__UBA184;f__UBA184;g__UBA184;s__UBA184sp030037695 | 66.33 | 1.20 |
| Bin 95 | d__Archaea;p__Thermoplasmatota;c__Thermoplasmata;o__UBA184;f__UBA184;g__UBA184;s__UBA184sp030039135 | 57.47 | 7.97 |
| Bin 25 | d__Archaea;p__Thermoplasmatota;c__Thermoplasmata;o__UBA184;f__UBA184;g__UBA184;s__UBA184sp030040755 | 93.06 | 2.40 |
| Bin 53 | d__Archaea;p__Thermoplasmatota;c__Thermoplasmata;o__UBA184;f__UBA184;g__UBA184;s__UBA184sp030044105 | 77.99 | 1.78 |
| Bin 51 | d__Archaea;p__Thermoproteota;c__Bathyarchaeia;o__40CM-2-53-6;f__FEN-987;g__;s__ | 78.67 | 4.75 |
| Bin 90 | d__Archaea;p__Thermoproteota;c__Bathyarchaeia;o__Bathyarchaeales;f__UBA233;g__Termiticorpusculum;s__ | 59.45 | 7.32 |
| Bin 114 | d__Archaea;p__Thermoproteota;c__Nitrososphaeria;o__Nitrososphaerales;f__CADDZS01;g__CADDZS01;s__ | 51.51 | 9.71 |
| Bin 11 | *d__Archaea;p__Thermoproteota;c__Nitrososphaeria;o__Nitrososphaerales;f__CADDZS01;g__CADDZS01;s__CADDZS01sp030041355* | *97.57* | *2.91* |
| Bin 22 | d__Archaea;p__Thermoproteota;c__Nitrososphaeria;o__Nitrososphaerales;f__Nitrosopumilaceae;g__Nitrosotalea;s__ | 95.45 | 0.19 |
| Bin 60 | d__Archaea;p__Thermoproteota;c__Nitrososphaeria;o__Nitrososphaerales;f__Nitrosopumilaceae;g__Nitrosotalea;s__Nitrosotaleasp030041535 | 73.30 | 5.31 |
| Bin 70 | d__Archaea;p__Thermoproteota;c__Nitrososphaeria;o__Nitrososphaerales;f__Nitrososphaeraceae;g__Nitrosopolaris;s__Nitrosopolarissp030038695 | 70.66 | 1.94 |
| Bin 39 | d__Archaea;p__Thermoproteota;c__Nitrososphaeria;o__Nitrososphaerales;f__UBA183;g__BOG-1369;s__ | 86.57 | 9.79 |
| Bin 116 | d__Archaea;p__Thermoproteota;c__Nitrososphaeria;o__Nitrososphaerales;f__UBA183;g__BOG-1369;s__ | 50.93 | 4.85 |
| Bin 31 | d__Archaea;p__Thermoproteota;c__Nitrososphaeria;o__Nitrososphaerales;f__UBA183;g__UBA183;s__ | 90.89 | 2.40 |
| Bin 112 | d__Archaea;p__Thermoproteota;c__Nitrososphaeria;o__Nitrososphaerales;f__UBA183;g__UBA183;s__ | 51.76 | 3.40 |
| Bin 98 | d__Bacteria;p__Acidobacteriota;c__Terriglobia;o__Acidoferrales;f__UBA7541;g__;s__ | 56.71 | 6.65 |
| Bin 97 | d__Bacteria;p__Acidobacteriota;c__Terriglobia;o__Acidoferrales;f__UBA7541;g__DAHUXN01;s__ | 56.78 | 6.84 |
| Bin 117 | d__Bacteria;p__Acidobacteriota;c__Terriglobia;o__Acidoferrales;f__UBA7541;g__DAHUXN01;s__DAHUXN01sp030037355 | 50.71 | 0.85 |
| Bin 69 | d__Bacteria;p__Acidobacteriota;c__Terriglobia;o__Acidoferrales;f__UBA7541;g__DAHUXN01;s__DAHUXN01sp030037775 | 71.13 | 2.85 |
| Bin 102 | d__Bacteria;p__Acidobacteriota;c__Terriglobia;o__Acidoferrales;f__UBA7541;g__JAAFGT01;s__ | 54.79 | 9.90 |
| Bin 57 | d__Bacteria;p__Acidobacteriota;c__Terriglobia;o__Acidoferrales;f__UBA7541;g__Palsa-295;s__ | 75.52 | 3.61 |
| Bin 36 | d__Bacteria;p__Acidobacteriota;c__Terriglobia;o__Terriglobales;f__Acidobacteriaceae;g__Acidobacterium;s__ | 89.80 | 9.59 |
| Bin 4 | d__Bacteria;p__Acidobacteriota;c__Terriglobia;o__Terriglobales;f__Acidobacteriaceae;g__Palsa-343;s__ | 99.57 | 3.36 |
| Bin 76 | d__Bacteria;p__Acidobacteriota;c__Terriglobia;o__Terriglobales;f__Acidobacteriaceae;g__Palsa-343;s__Palsa-343sp030038115 | 67.48 | 3.63 |
| Bin 82 | d__Bacteria;p__Acidobacteriota;c__Terriglobia;o__Terriglobales;f__Acidobacteriaceae;g__Palsa-343;s__Palsa-343sp030038115 | 63.97 | 1.72 |
| Bin 62 | d__Bacteria;p__Acidobacteriota;c__Terriglobia;o__Terriglobales;f__SbA1;g__JAFAGX01;s__ | 72.39 | 4.27 |
| Bin 66 | d__Bacteria;p__Acidobacteriota;c__Terriglobia;o__Terriglobales;f__SbA1;g__JAFAGX01;s__ | 71.51 | 0.85 |
| Bin 5 | d__Bacteria;p__Acidobacteriota;c__Terriglobia;o__Terriglobales;f__SbA1;g__QIAJ01;s__ | 99.15 | 3.42 |
| Bin 14 | d__Bacteria;p__Acidobacteriota;c__Terriglobia;o__Terriglobales;f__SbA1;g__QIAJ01;s__ | 96.74 | 4.56 |
| Bin 20 | d__Bacteria;p__Acidobacteriota;c__Terriglobia;o__Terriglobales;f__SbA1;g__QIAJ01;s__ | 95.84 | 9.69 |
| Bin 8 | d__Bacteria;p__Acidobacteriota;c__Terriglobia;o__Terriglobales;f__SbA1;g__Sulfotelmatobacter;s__ | 98.67 | 5.13 |
| Bin 120 | d__Bacteria;p__Acidobacteriota;c__Terriglobia;o__Terriglobales;f__SbA1;g__Sulfotelmatobacter;s__Sulfotelmatobactersp030038255 | 50.31 | 0.00 |
| Bin 105 | d__Bacteria;p__Acidobacteriota;c__Terriglobia;o__Terriglobales;f__SbA1;g__Sulfotelmatobacter;s__Sulfotelmatobactersp030039165 | 54.04 | 9.83 |
| Bin 48 | d__Bacteria;p__Acidobacteriota;c__Terriglobia;o__UBA7540;f__UBA7540;g__UBA7540;s__ | 80.70 | 3.42 |
| Bin 91 | d__Bacteria;p__Actinomycetota;c__Acidimicrobiia;o__Acidimicrobiales;f__Palsa-688;g__Fen-671;s__ | 58.87 | 4.59 |
| Bin 16 | d__Bacteria;p__Actinomycetota;c__Acidimicrobiia;o__Acidimicrobiales;f__RAAP-2;g__RAAP-2;s__ | 96.58 | 3.85 |
| Bin 17 | d__Bacteria;p__Actinomycetota;c__Acidimicrobiia;o__Acidimicrobiales;f__RAAP-2;g__RAAP-2;s__ | 96.15 | 2.64 |
| Bin 43 | d__Bacteria;p__Actinomycetota;c__Acidimicrobiia;o__Acidimicrobiales;f__RAAP-2;g__RAAP-2;s__ | 83.79 | 5.98 |
| Bin 12 | d__Bacteria;p__Actinomycetota;c__Acidimicrobiia;o__Acidimicrobiales;f__UBA8190;g__UBA8190;s__ | 97.25 | 1.28 |
| Bin 103 | d__Bacteria;p__Actinomycetota;c__Acidimicrobiia;o__Acidimicrobiales;f__UBA8190;g__UBA8190;s__ | 54.70 | 7.07 |
| Bin 15 | d__Bacteria;p__Actinomycetota;c__Actinomycetes;o__Streptosporangiales;f__Streptosporangiaceae;g__JAJYUU01;s__JAJYUU01sp030038715 | 96.58 | 5.19 |
| Bin 65 | d__Bacteria;p__Actinomycetota;c__Actinomycetes;o__Streptosporangiales;f__Streptosporangiaceae;g__Palsa-504;s__ | 71.62 | 6.76 |
| Bin 67 | d__Bacteria;p__Bacillota_E;c__JAJZYS01;o__JAJZYS01;f__JAILZS01;g__JAILZS01;s__ | 71.29 | 8.12 |
| Bin 68 | d__Bacteria;p__Bacteroidota;c__Bacteroidia;o__AKYH767;f__Palsa-948_A;g__JABEUT01;s__ | 71.17 | 0.35 |
| Bin 101 | d__Bacteria;p__Bdellovibrionota;c__Bdellovibrionia;o__Bdellovibrionales;f__UBA6776;g__;s__ | 54.88 | 1.48 |
| Bin 41 | d__Bacteria;p__Chlamydiota;c__Chlamydiia;o__Chlamydiales;f__CAIPRZ01;g__;s__ | 86.16 | 0.80 |
| Bin 108 | d__Bacteria;p__Dependentiae;c__Babeliae;o__Babeliales;f__Vermiphilaceae;g__;s__ | 53.66 | 1.26 |
| Bin 42 | d__Bacteria;p__Desulfobacterota_B;c__Binatia;o__Binatales;f__Binataceae;g__;s__ | 85.93 | 3.12 |
| Bin 94 | d__Bacteria;p__Desulfobacterota_B;c__Binatia;o__Binatales;f__Binataceae;g__JAFAHS01;s__JAFAHS01sp030041155 | 57.87 | 9.75 |
| Bin 72 | d__Bacteria;p__Elusimicrobiota;c__Elusimicrobia;o__UBA1565;f__UBA9628;g__;s__ | 68.94 | 0.25 |
| Bin 73 | d__Bacteria;p__Elusimicrobiota;c__Elusimicrobia;o__UBA1565;f__UBA9628;g__;s__ | 68.94 | 1.52 |
| Bin 18 | d__Bacteria;p__Eremiobacterota;c__Eremiobacteria;o__Baltobacterales;f__Baltobacteraceae;g__Tyrphobacter;s__ | 95.99 | 6.43 |
| Bin 58 | d__Bacteria;p__FCPU426;c__Palsa-1180;o__Palsa-1180;f__Palsa-1180;g__;s__ | 74.91 | 1.56 |
| Bin 100 | d__Bacteria;p__FCPU426;c__Palsa-1180;o__Palsa-1180;f__Palsa-1180;g__;s__ | 55.21 | 8.34 |
| Bin 19 | d__Bacteria;p__FCPU426;c__Palsa-1180;o__Palsa-1180;f__Palsa-1180;g__JAJPJW01;s__ | 95.85 | 5.59 |
| Bin 38 | d__Bacteria;p__FCPU426;c__Palsa-1180;o__Palsa-1180;f__Palsa-1180;g__JAJPJW01;s__ | 87.53 | 4.93 |
| Bin 2 | d__Bacteria;p__Nitrospirota_A;c__Leptospirillia;o__Leptospirillales;f__Leptospirillaceae;g__;s__ | 99.98 | 0.28 |
| Bin 80 | d__Bacteria;p__Nitrospirota_A;c__Leptospirillia;o__Leptospirillales;f__Leptospirillaceae;g__JAJYGC01;s__ | 66.12 | 0.06 |
| Bin 113 | d__Bacteria;p__Nitrospirota;c__Thermodesulfovibrionia;o__Thermodesulfovibrionales;f__Dissulfurispiraceae;g__JADFXI01;s__ | 51.65 | 9.95 |
| Bin 54 | d__Bacteria;p__Patescibacteria;c__Paceibacteria;o__UBA9983_A;f__GWA1-40-21;g__Bog-1340;s__ | 77.78 | 2.18 |
| Bin 23 | d__Bacteria;p__Pseudomonadota;c__Alphaproteobacteria;o__Acetobacterales;f__Acetobacteraceae;g__Acidocella;s__ | 94.25 | 6.70 |
| Bin 110 | d__Bacteria;p__Pseudomonadota;c__Alphaproteobacteria;o__Acetobacterales;f__Acetobacteraceae;g__JAJZVS01;s__ | 52.15 | 0.43 |
| Bin 24 | d__Bacteria;p__Pseudomonadota;c__Alphaproteobacteria;o__Acetobacterales;f__Acetobacteraceae;g__JAKAWQ01;s__JAKAWQ01sp030037175 | 93.33 | 2.90 |
| Bin 50 | d__Bacteria;p__Pseudomonadota;c__Alphaproteobacteria;o__ATCC43930;f__Stellaceae;g__;s__ | 79.31 | 6.90 |
| Bin 64 | d__Bacteria;p__Pseudomonadota;c__Alphaproteobacteria;o__ATCC43930;f__Stellaceae;g__;s__ | 71.63 | 0.77 |
| Bin 55 | d__Bacteria;p__Pseudomonadota;c__Alphaproteobacteria;o__ATCC43930;f__Stellaceae;g__AP-15;s__ | 76.83 | 7.41 |
| Bin 88 | d__Bacteria;p__Pseudomonadota;c__Alphaproteobacteria;o__ATCC43930;f__Stellaceae;g__AP-15;s__ | 60.85 | 7.14 |
| Bin 93 | d__Bacteria;p__Pseudomonadota;c__Alphaproteobacteria;o__ATCC43930;f__Stellaceae;g__AP-15;s__ | 58.44 | 2.23 |
| Bin 106 | d__Bacteria;p__Pseudomonadota;c__Alphaproteobacteria;o__ATCC43930;f__Stellaceae;g__AP-15;s__ | 54.04 | 7.22 |
| Bin 35 | d__Bacteria;p__Pseudomonadota;c__Alphaproteobacteria;o__ATCC43930;f__Stellaceae;g__AP-15;s__AP-15sp030041385 | 90.03 | 6.06 |
| Bin 78 | d__Bacteria;p__Pseudomonadota;c__Alphaproteobacteria;o__ATCC43930;f__Stellaceae;g__AP-15;s__AP-15sp030042035 | 66.93 | 8.62 |
| Bin 40 | d__Bacteria;p__Pseudomonadota;c__Alphaproteobacteria;o__Rhizobiales;f__Beijerinckiaceae;g__Methylocystis;s__ | 86.52 | 4.73 |
| Bin 81 | d__Bacteria;p__Pseudomonadota;c__Alphaproteobacteria;o__Rhizobiales;f__Beijerinckiaceae;g__Methylocystis;s__ | 65.27 | 3.61 |
| Bin 107 | d__Bacteria;p__Pseudomonadota;c__Alphaproteobacteria;o__Rhizobiales;f__Beijerinckiaceae;g__Methylocystis;s__ | 53.74 | 6.03 |
| Bin 85 | d__Bacteria;p__Pseudomonadota;c__Alphaproteobacteria;o__Rhizobiales;f__Beijerinckiaceae;g__Methylovirgula;s__Methylovirgulasp030044485 | 62.56 | 7.46 |
| Bin 37 | d__Bacteria;p__Pseudomonadota;c__Alphaproteobacteria;o__Rhizobiales;f__Beijerinckiaceae;g__Rhodoblastus;s__ | 88.05 | 8.08 |
| Bin 115 | d__Bacteria;p__Pseudomonadota;c__Alphaproteobacteria;o__Rhizobiales;f__Xanthobacteraceae;g__BOG-931;s__ | 51.16 | 9.74 |
| Bin 52 | d__Bacteria;p__Pseudomonadota;c__Alphaproteobacteria;o__Rhizobiales;f__Xanthobacteraceae;g__BOG-931;s__BOG-931sp030038415 | 78.07 | 7.65 |
| Bin 7 | d__Bacteria;p__Pseudomonadota;c__Alphaproteobacteria;o__Sphingomonadales;f__Sphingomonadaceae;g__Novosphingobium;s__ | 98.91 | 1.13 |
| Bin 10 | d__Bacteria;p__Pseudomonadota;c__Alphaproteobacteria;o__Sphingomonadales;f__Sphingomonadaceae;g__Novosphingobium;s__ | 97.65 | 5.11 |
| Bin 49 | d__Bacteria;p__Pseudomonadota;c__Gammaproteobacteria;o__Berkiellales;f__Berkiellaceae;g__;s__ | 79.73 | 4.65 |
| Bin 86 | d__Bacteria;p__Pseudomonadota;c__Gammaproteobacteria;o__Burkholderiales;f__Burkholderiaceae;g__GJ-E10;s__ | 62.54 | 9.34 |
| Bin 32 | d__Bacteria;p__Pseudomonadota;c__Gammaproteobacteria;o__Burkholderiales;f__Burkholderiaceae;g__Paraburkholderia;s__ | 90.66 | 0.53 |
| Bin 46 | d__Bacteria;p__Pseudomonadota;c__Gammaproteobacteria;o__Burkholderiales;f__Burkholderiaceae;g__Paraburkholderia;s__ | 82.03 | 7.56 |
| Bin 77 | d__Bacteria;p__Pseudomonadota;c__Gammaproteobacteria;o__Burkholderiales;f__Burkholderiaceae;g__Paraburkholderia;s__ | 67.07 | 0.00 |
| Bin 6 | d__Bacteria;p__Pseudomonadota;c__Gammaproteobacteria;o__Burkholderiales;f__Burkholderiaceae;g__PKWO01;s__ | 98.94 | 0.19 |
| Bin 3 | d__Bacteria;p__Pseudomonadota;c__Gammaproteobacteria;o__Burkholderiales;f__Burkholderiaceae;g__Thiomonas;s__ | 99.57 | 0.10 |
| Bin 9 | d__Bacteria;p__Pseudomonadota;c__Gammaproteobacteria;o__Burkholderiales;f__Chromobacteriaceae;g__Aquitalea;s__ | 98.44 | 0.41 |
| Bin 61 | d__Bacteria;p__Pseudomonadota;c__Gammaproteobacteria;o__Burkholderiales;f__SG8-41;g__;s__ | 72.40 | 3.09 |
| Bin 119 | d__Bacteria;p__Pseudomonadota;c__Gammaproteobacteria;o__JABCZS01;f__JABCZS01;g__;s__ | 50.44 | 3.00 |
| Bin 29 | d__Bacteria;p__Pseudomonadota;c__Gammaproteobacteria;o__Pseudomonadales;f__Moraxellaceae;g__;s__ | 91.59 | 3.65 |
| Bin 47 | d__Bacteria;p__Pseudomonadota;c__Gammaproteobacteria;o__Steroidobacterales;f__Steroidobacteraceae;g__13-2-20CM-66-19;s__ | 80.89 | 4.62 |
| Bin 28 | d__Bacteria;p__Pseudomonadota;c__Gammaproteobacteria;o__Steroidobacterales;f__Steroidobacteraceae;g__Bog-1198;s__ | 92.21 | 8.39 |
| Bin 1 | d__Bacteria;p__Pseudomonadota;c__Gammaproteobacteria;o__UBA12402;f__UBA12402;g__;s__ | 100.00 | 0.21 |
| Bin 44 | d__Bacteria;p__Pseudomonadota;c__Gammaproteobacteria;o__Xanthomonadales;f__Rhodanobacteraceae;g__Dyella_B;s__ | 83.32 | 6.40 |
| Bin 111 | d__Bacteria;p__Pseudomonadota;c__Gammaproteobacteria;o__Xanthomonadales;f__Rhodanobacteraceae;g__Dyella_B;s__ | 51.95 | 1.72 |
| Bin 104 | d__Bacteria;p__Pseudomonadota;c__Gammaproteobacteria;o__Xanthomonadales;f__Rhodanobacteraceae;g__Rhodanobacter;s__ | 54.12 | 8.10 |
| Bin 118 | d__Bacteria;p__Verrucomicrobiota;c__Verrucomicrobiae;o__Limisphaerales;f__Palsa-1400;g__Palsa-1400;s__ | 50.45 | 3.82 |
| Bin 74 | d__Bacteria;p__Verrucomicrobiota;c__Verrucomicrobiae;o__Pedosphaerales;f__UBA11358;g__UBA7542;s__ | 68.78 | 2.79 |

**Supplementary Figure.1: Ratio of *δ*^13^C-CO_2_ and *δ*^13^C-CH_4_ in the lower depths show that the carbon in non amened setup gets oxidised with time compared to the Fe amended setup.**

(a)

(b)

(c)

**Supplementary Figure.2: Box plot representing changes in the intensity of the wave number across different depths and treatments. (a) Upper, (b) middle, and (c) lower depth peat sample under different treatments. Light blue indicates day 0 samples (OS), grey indicates Fe-amended peat setup at day 42, and dark green indicates unamended peat (noFe) setup at day 42.**

| (a) |  |
| --- | --- |
| (b) |  |
| (c) |  |

**Supplementary Figure.3: Metabolite profiles change on treatment with Fe of (a)Carbonyl compounds, esters, fatty acids and derivatives, amides, organic acids and derivatives, ketal, Ethers, amino acids, peptides, and analogs, lipids, steroidal glycosides, phosphates, sulfur compounds, amino acids, peptides, (b)alcohols, alkaloids, amines, carbohydrates and derivatives, benzoic acids, phenolic compounds, sugar derivatives, carboxylic acids, glycosides, amino sugars, fatty alcohols, (c)organophosphorus compounds, monoterpenoids, isoquinolines, guanidines, pyrimidines, sulfur compounds, methane monoterpenoids, nitrosamines, silanes, hopanoids, lactones and triterpenoids.**

**Supplementary Figure 4: Changes in abundance of archaeal phyla across depths and treatment.**

|  |  |
| --- | --- |

**Supplementary Figure.5: Changes in the relative abundance of major archaeal classes.**

**Supplementary Figure 6: The Spearman correlation of the top 23 genera plotted over time with treatment specific linear trend lines.**

| Upper | Middle | Lower |
| --- | --- | --- |
|  |  |  |

**Supplementary Figure 7: Volcano plot representing the differentially enriched genes (q value < 0.05) involved in sulfur metabolism, nitrogen metabolism, C1, metabolism, carbon degradation, stress response, hydrogenases, fermentation, and Wood-ljungdahl pathways.**

**Supplementary Figure.7 Gene presence and metabolic pathways that are present**

**Supplementary Figure 8: Key Cazymes involved in the degradation of degradation of carbon from metagenome.**

**References:**

1.	Viollier E, Inglett PW, Hunter K, Roychoudhury AN, Van Cappellen P. The ferrozine method revisited: Fe(II)/Fe(III) determination in natural waters. Applied Geochemistry. 2000;15(6):785-90.

2.	Pavagadhi S, Benke PI, Majedi SM, Umashankar S, Yeap YT, Swarup S. Chapter 16 - Evaluating the effects of environmental perturbations in bloom forming cyanobacteria through untargeted metabolomics. In: Beale DJ, Hillyer KE, Warden AC, Jones OAH, editors. Applied Environmental Metabolomics: Academic Press; 2022. p. 231-44.

3.	Parada AE, Needham DM, Fuhrman JA. Every base matters: assessing small subunit rRNA primers for marine microbiomes with mock communities, time series and global field samples. Environmental Microbiology. 2016;18(5):1403-14.

4.	Callahan BJ, McMurdie PJ, Rosen MJ, Han AW, Johnson AJA, Holmes SP. DADA2: High-resolution sample inference from Illumina amplicon data. Nat Methods. 2016;13(7):581-3.

5.	Davis NM, Proctor DM, Holmes SP, Relman DA, Callahan BJ. Simple statistical identification and removal of contaminant sequences in marker-gene and metagenomics data. Microbiome. 2018;6(1):226.

6.	McMurdie PJ, Holmes S. phyloseq: an R package for reproducible interactive analysis and graphics of microbiome census data. PLoS One. 2013;8(4):e61217.

7.	William H. ggplot2: Elegant Graphics for Data Analysis. Springer-Verlag New York; 2016.

8.	Oksanen J, Simpson G, Blanchet F, Kindt R, Legendre P, Minchin P, et al. vegan: Community Ecology Package. 2024.

9.	Neuwirth E. RColorBrewer: ColorBrewer Palettes. 2022.

10.	Kassambara A. ggpubr: 'ggplot2' Based Publication Ready Plots. 2018.

11.	Wei T, Simko V. R package 'corrplot': Visualization of a Correlation Matrix. 2024.

12.	Kolde R. pheatmap: Pretty Heatmaps. 2019.

13.	Martin M. Cutadapt removes adapter sequences from high-throughput sequencing reads. EMBnetjournal; Vol 17, No 1: Next Generation Sequencing Data Analysis. 2011.

14.	Bushnell B, editor BBMap: A Fast, Accurate, Splice-Aware Aligner2014 2014-03-17; United States. Research Org.: Lawrence Berkeley National Lab. (LBNL), Berkeley, CA (United States)Sponsor Org.: USDOE Office of Science (SC).

15.	Bandla A, Mukhopadhyay S, Mishra S, Sudarshan AS, Swarup S. Genome-resolved carbon processing potential of tropical peat microbiomes from an oil palm plantation. Scientific Data. 2023;10(1):373.

16.	Bandla A, Pavagadhi S, Sridhar Sudarshan A, Poh MCH, Swarup S. 910 metagenome-assembled genomes from the phytobiomes of three urban-farmed leafy Asian greens. Scientific Data. 2020;7(1):278.

17.	Nurk S, Meleshko D, Korobeynikov A, Pevzner PA. metaSPAdes: a new versatile metagenomic assembler. Genome Res. 2017;27(5):824-34.

18.	Li D, Liu C-M, Luo R, Sadakane K, Lam T-W. MEGAHIT: an ultra-fast single-node solution for large and complex metagenomics assembly via succinct de Bruijn graph. Bioinformatics. 2015;31(10):1674-6.

19.	Gurevich A, Saveliev V, Vyahhi N, Tesler G. QUAST: quality assessment tool for genome assemblies. Bioinformatics. 2013;29(8):1072-5.

20.	Kang DD, Li F, Kirton E, Thomas A, Egan R, An H, et al. MetaBAT 2: an adaptive binning algorithm for robust and efficient genome reconstruction from metagenome assemblies. PeerJ. 2019;7:e7359.

21.	Wu Y-W, Simmons BA, Singer SW. MaxBin 2.0: an automated binning algorithm to recover genomes from multiple metagenomic datasets. Bioinformatics. 2016;32(4):605-7.

22.	Chklovski A, Parks DH, Woodcroft BJ, Tyson GW. CheckM2: a rapid, scalable and accurate tool for assessing microbial genome quality using machine learning. Nat Methods. 2023;20(8):1203-12.

23.	Sieber CMK, Probst AJ, Sharrar A, Thomas BC, Hess M, Tringe SG, et al. Recovery of genomes from metagenomes via a dereplication, aggregation and scoring strategy. Nature Microbiology. 2018;3(7):836-43.

24.	Olm MR, Brown CT, Brooks B, Banfield JF. dRep: a tool for fast and accurate genomic comparisons that enables improved genome recovery from metagenomes through de-replication. The ISME Journal. 2017;11(12):2864-8.

25.	Chaumeil PA, Mussig AJ, Hugenholtz P, Parks DH. GTDB-Tk v2: memory friendly classification with the genome taxonomy database. Bioinformatics. 2022;38(23):5315-6.

26.	Zhou Z, Tran PQ, Breister AM, Liu Y, Kieft K, Cowley ES, et al. METABOLIC: high-throughput profiling of microbial genomes for functional traits, metabolism, biogeochemistry, and community-scale functional networks. Microbiome. 2022;10(1):33.

27.	Aroney STN, Newell RJP, Nissen JN, Camargo AP, Tyson GW, Woodcroft BJ. CoverM: read alignment statistics for metagenomics. Bioinformatics. 2025;41(4):btaf147.

28.	Asnicar F, Thomas AM, Beghini F, Mengoni C, Manara S, Manghi P, et al. Precise phylogenetic analysis of microbial isolates and genomes from metagenomes using PhyloPhlAn 3.0. Nature Communications. 2020;11(1):2500.

29.	Letunic I, Bork P. Interactive Tree Of Life (iTOL) v5: an online tool for phylogenetic tree display and annotation. Nucleic Acids Res. 2021;49(W1):W293-W6.

30.	Seemann T. Prokka: rapid prokaryotic genome annotation. Bioinformatics. 2014;30(14):2068-9.

31.	Mallick H, Rahnavard A, McIver LJ, Ma S, Zhang Y, Nguyen LH, et al. Multivariable association discovery in population-scale meta-omics studies. PLOS Computational Biology. 2021;17(11):e1009442.
